# Decomposing and Tracing Mutual Information by Quantifying Reachable Decision Regions

**DOI:** 10.3390/e25071014

**Published:** 2023-06-30

**Authors:** Tobias Mages, Christian Rohner

**Affiliations:** Department of Information Technology, Uppsala University, 752 36 Uppsala, Sweden; christian.rohner@it.uu.se

**Keywords:** partial information decomposition, redundancy, synergy, information flow analysis

## Abstract

The idea of a partial information decomposition (PID) gained significant attention for attributing the components of mutual information from multiple variables about a target to being unique, redundant/shared or synergetic. Since the original measure for this analysis was criticized, several alternatives have been proposed but have failed to satisfy the desired axioms, an inclusion–exclusion principle or have resulted in negative partial information components. For constructing a measure, we interpret the achievable type I/II error pairs for predicting each state of a target variable (reachable decision regions) as notions of pointwise uncertainty. For this representation of uncertainty, we construct a distributive lattice with mutual information as consistent valuation and obtain an algebra for the constructed measure. The resulting definition satisfies the original axioms, an inclusion–exclusion principle and provides a non-negative decomposition for an arbitrary number of variables. We demonstrate practical applications of this approach by tracing the flow of information through Markov chains. This can be used to model and analyze the flow of information in communication networks or data processing systems.

## 1. Introduction

A Partial Information Decomposition (PID) aims to attribute the provided information about a discrete target variable *T* from a set of predictor or viewable variables $V=\{V_{1},\ldots ,V_{n}\}$ to each individual variable $V_{i}$. The partial contributions to the information about *T* may be provided by all variables (redundant or shared), by a specific variable (unique) or only be available through a combination of variables (synergetic/complementing) [1]. This decomposition is particularly applicable when studying complex systems. For example, it can be used to study logical circuits, neural networks [2] or the propagation of information over multiple paths through a network. The concept of synergy has been applied to develop data privacy techniques [3,4], and we think that the concept of redundancy may be suitable to study a notion of robustness in data processing systems.

Unfortunately and to the best of our knowledge, there does not exist a non-negative decomposition of mutual information for an arbitrary number of variables that satisfies the commutativity, monotonicity and self-redundancy axioms except the original measure of Williams and Beer [5]. However, this measure has been criticized for not distinguishing “the *same* information and the *same amount* of information” [6,7,8,9].

**Figure 1:**
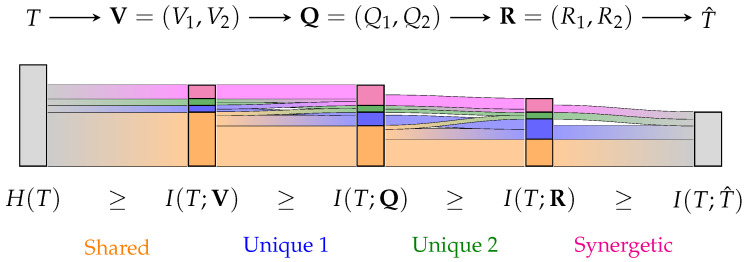
Visualization of a partial information decomposition with information flow analysis of a Markov chain as Sankey diagram. A partial information decomposition enables attributing the provided information about *T* to being shared (**orange**), unique (**blue/green**) or synergetic/complementing (**pink**). While this already offers practical insights for studying complex systems, the ability to trace the flow of partial information may create a valuable tool to model and analyze many applications.

Here, we propose an alternative non-negative partial information decomposition that satisfies Williams and Beer’s axioms [5] for an arbitrary number of variables. It provides an intuitive operational interpretation and results in an algebra like probability theory. To demonstrate that the approach distinguishes the same information from the same amount of information, we highlight its application in tracing the flow of information through a Markov chain, as visualized in Figure 1.

This work is structured in three parts: Section 2 provides an overview of the related work and background information. Section 3 presents a representation of pointwise uncertainty, constructs a distributive lattice and demonstrates that mutual information is the expected value of its consistent valuation. Section 4 discusses applications of the resulting measure to PIDs and the tracing of information through Markov chains. We provide an overview of the used notation at the end of the paper.

## 2. Related Work

We briefly summarize partial orders and the four main publications which led to our proposed decomposition approach. This includes the PID by Williams and Beer [5], the quantification of unique information by Bertschinger et al. [10] and Griffith and Koch [11], the Blackwell order based on Bertschinger and Rauh [12], the evaluation of binary decision problems using Receiver Operating Characteristics and consistent lattice valuations by Knuth [13].

### 2.1. Partial Orders and Lattices

This section provides a brief overview of the relevant definitions on partial orders and lattices for the context of this work based on [9,13]. A binary ordering relation ≼ on a set L is called a *preorder* if it is reflexive and transitive. If the ordering relation additionally satisfies an antisymmetry, then (L,≼) is called a *partially ordered set* (*poset*). For α,β,γ∈L:
α≼α(reflexivity)ifα≼βandβ≼γthenα≼γ(transitivity)ifα≼βandβ≼αthenα=β     (antisymmetry)

Two elements satisfy α≼β, β≼α or may be incomparable, meaning α⋠β and β⋠α. A partially ordered set has a *bottom element* ⊥∈L if ⊥≼α for all α∈L and a *top element* ⊤∈L if α≼⊤ for all α∈L. For each element α, it can be defined a down-set (↓α) and up-set (↑α) as well as a strict down-set ($\dot{↓}\alpha$) and strict up-set ($\dot{↑}\alpha$) as shown below:

$$\begin{array}{ccc} ↓\alpha & =\{\beta \in L|\beta ≼\alpha \} & \left(\mathrm{down}\text{-}\mathrm{set}\right)\;\\ \;\dot{↓}\alpha & =\{\beta \in L|\beta ≼\alpha \mathrm{and}\alpha ⋠\beta \} & \;\left(\mathrm{strict}\mathrm{down}\text{-}\mathrm{set}\right)\;\\ ↑\alpha & =\{\beta \in L|\alpha ≼\beta \} & \left(\mathrm{up}\text{-}\mathrm{set}\right)\;\\ \dot{↑}\alpha & =\{\beta \in L|\alpha ≼\beta \mathrm{and}\beta ⋠\alpha \} & \left(\mathrm{strict}\mathrm{up}\text{-}\mathrm{set}\right) \end{array}$$

A *lattice* is a partially ordered set (L,≼) for which every pair of elements {α,b}⊆L has a unique least upper bound α⋎β=sup{α,β}, referred to as *joint*, and a unique greatest lower bound α⋏β=inf{α,β}, referred to as *meet*. This creates an algebra (L,⋏,⋎) with the binary operators ⋏ and ⋎ that satisfies indempotency, commutativity, associativity and absorption. The consistency relates the ordering relation and algebra with each other. A *distributive lattice* additionally satisfies distributivity.
α⋎α=αα⋏α=α(indempotency)α⋎β=β⋎αα⋏β=β⋏α(commutativity)α⋎(β⋎γ)=(α⋎β)⋎γα⋏(β⋏γ)=(α⋏β)⋏γ(associativity)α⋎(α⋏β)=αα⋏(α⋎β)=α(absorption)α≼β⇒α⋎β=βα≼β⇒α⋏β=α(consistency)α⋎(β⋏γ)=(α⋎β)⋏(α⋎γ) α⋏(β⋎γ)=(α⋏β)⋎(α⋏γ)(distributivity)

### 2.2. Partial Information Decomposition

This section summarizes Williams and Beer’s general approach to partial information decompositions [5]. A more detailed discussion of the literature and required background can be found in [9] (pp. 6–20).

**Figure 2:**
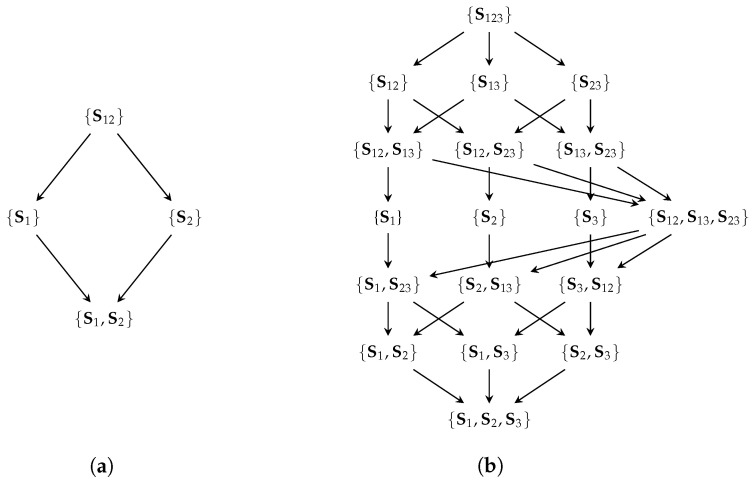
The redundancy lattices for two (**a**) and three (**b**) visible variables. The redundancy lattice specifies the expected inclusion relation between atoms. The following function $I_{\cap }$ shall measure the shared information for a sets of variables such that the element $\left\{S_{1},S_{2}\right\}$ represents the shared information between $S_{1}$ and $S_{2}$ about the target variable *T*.

Williams and Beer [5] define *sources* $S_{i}\in P_{1}\left(V\right)$ as all combinations of viewable variables ($P_{1}\left(V\right)$ referring to the power set of V without the empty set) and use Equation (1a) to construct all distinct interactions between them α∈A(V), which are referred to as *partial information atoms*. Equation (1b) provides a partial order of atoms to construct a *redundancy lattice* (A(V),≼). As a convention, we indicate the visible variables contained in a source by its index, such as $S_{12}=\left\{V_{1},V_{2}\right\}$. The example of the redundancy lattice for two and three visible variables is shown in Figure 2.

(1a) $$\begin{array}{c} A\left(V\right)=\{\alpha \in P_{1}\left(P_{1}\left(V\right)\right)|\forall S_{i},S_{j}\in \alpha ,S_{i}⊄S_{j}\}, \end{array}$$

(1b) $$\begin{array}{c} \forall \alpha ,\beta \in A\left(V\right),\left(\alpha ≼\beta \Leftrightarrow \forall S_{j}\in \beta ,\exists S_{i}\in \alpha |S_{i}\subseteq S_{j}\right). \end{array}$$

A measure of *redundant information* $I_{\cap }$ shall be defined for this lattice as “[*…*] cumulative information function which in effect integrates the contribution from each node as one moves up through the nodes of the lattice” [9] (p. 15). Williams and Beer [5] then use the Möbius inverse (Equation (2)) to identify the *partial information* $I_{\delta }\left(\alpha ;T\right)$ as the contribution of atom α∈A(V) and therefore the desired unique/redundant/synergetic component. A PID is said to be *non-negative* if the resulting partial contributions are guaranteed to be non-negative.

(2) $$I_{\delta }\left(\alpha ;T\right)=I_{\cap }\left(\alpha ;T\right)-\sum_{\beta \in \dot{↓}\alpha }I_{\delta }\left(\beta ;T\right).$$

Williams and Beer [5] highlight three axioms that a measure of redundancy should satisfy.

**Axiom** **1** (Commutativity)**.** *Invariant to the order of sources (σ permuting the order of indices):*

$$I_{\cap }\left(S_{1},\ldots ,S_{i};T\right)=I_{\cap }\left(S_{\sigma (1)},\ldots ,S_{\sigma (i)};T\right)$$

**Axiom** **2** (Monotonicity)**.** *Additional sources can only decrease redundant information:*

$$I_{\cap }\left(S_{1},\ldots ,S_{i-1};T\right)\geq I_{\cap }\left(S_{1},\ldots ,S_{i};T\right)$$

**Axiom** **3** (Self-redundancy)**.** *For a single source, redundancy equals mutual information:*

$$I_{\cap }\left(S_{i};T\right)=I\left(S_{i};T\right)$$

Finally, Williams and Beer [5] proposed $I_{\min}$ (Equation (3)) as a measure of redundancy and demonstrated that it satisfies the required axioms.

(3a) $$\begin{array}{cc} I(S_{i};T=t) & =\sum_{s\in S_{i}}p\left(s|t\right)\left[\log\frac{1}{p(t)}-\log\frac{1}{p(t|s)}\right] \end{array}$$

(3b) $$\begin{array}{cc} I_{\min}\left(S_{1},\ldots ,S_{k};T\right) & =\sum_{t\in T}p\left(t\right)\min_{i\in 1..k}I\left(S_{i};T=t\right). \end{array}$$

However, the measure has been criticized for not distinguishing “the *same* information and the *same amount* of information” [6,7,8,9] due to its use of a pointwise minimum (for each t∈T) over the sources.

### 2.3. Quantifying Unique Information

A non-negative decomposition for the case of two viewable variables $V=\{V_{1},V_{2}\}$ was proposed by Bertschinger et al. [10] (defining unique information) as well as an equivalent decomposition by Griffith and Koch [11] (defining union information) as shown in Equation (4) (modified notation). The function $\vartheta (V_{1},V_{2};T)$ acts as an information measure of the union for $V_{1}$ and $V_{2}$ (the minimal information that any two variables with the same marginal distributions can achieve), which is then used to compute the partial contributions using an inclusion–exclusion principle. Bertschinger et al. [10] motivated the decomposition from the operational interpretation that if a variable provides unique information, there must be a way to utilize this information in a decision problem for some reward function. Additionally, they argue that unique information should only depend on the marginal distributions $P_{\left(T,V_{1}\right)}$ and $P_{\left(T,V_{2}\right)}$.

(4) $$\begin{array}{ccc} \vartheta (V_{1},V_{2};T)= & \min\;I\left(F;G_{1},G_{2}\right)\;s.t.\;P_{\left(F,G_{1}\right)}=P_{\left(T,V_{1}\right)}\;\mathrm{and}\;P_{\left(F;G_{2}\right)}=P_{\left(T,V_{2}\right)}\\ S(V_{1},V_{2};T) & =I\left(V_{1};T\right)+I\left(V_{2};T\right)-\vartheta \left(V_{1},V_{2};T\right) & \left(\mathrm{Shared}\right)\\ U(V_{1};T)\;\; & =\vartheta \left(V_{1},V_{2};T\right)-I\left(V_{1};T\right) & \left(\mathrm{Unique}\right)\\ C(V_{1},V_{2};T) & =I\left(T;V_{1},V_{2}\right)-\vartheta \left(V_{1},V_{2};T\right) & \left(\mathrm{Complementing}\right) \end{array}$$

We highlight this decomposition since our approach can be interpreted as its pointwise extension (see Section 4.1).

### 2.4. Blackwell Order

**Figure 3:**
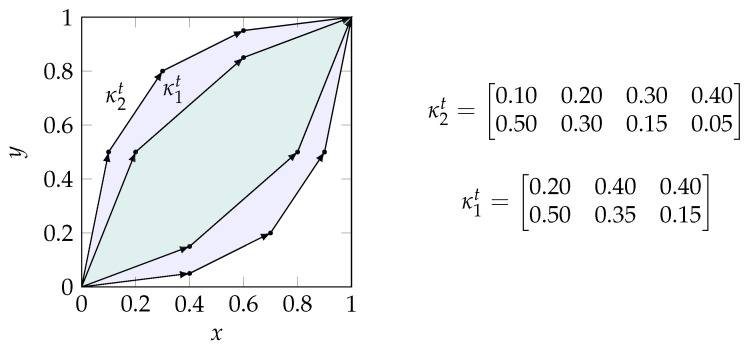
Visualization of the zonotope order for binary input channels. The channel $\kappa_{1}^{t}$ is Blackwell inferior to $\kappa_{2}^{t}$ ($\kappa_{1}^{t}⊑\kappa_{2}^{t}$) since the corresponding zonotope $Z_{\kappa_{1}^{t}}$ (**green**) is a subset of $Z_{\kappa_{2}^{t}}$ (**purple**). As a result, the meet and joint elements of this example are: $\kappa_{1}^{t}⊓\kappa_{2}^{t}=\kappa_{1}^{t}$ and $\kappa_{1}^{t}⊔\kappa_{2}^{t}=\kappa_{2}^{t}$.

A channel κ can be represented as a (row) stochastic matrix wherein each element is non-negative and all rows sum to one (see Figure 3). In this work, we consider the sources to be the indirect observation of the target variable through a channel $T\overset{\kappa_{i}}{\to }S_{i}$ while taking the joint distribution of the visible variables within $S_{i}$. As a result, $\kappa_{i}$ is obtained from the conditional probability distribution $\kappa_{i}=T\to S_{i}=P_{\left(S_{i}|T\right)}$. As for sources, we list the contained visible variables as an index such that i=12 corresponds to $\kappa_{12}=P_{\left(S_{12}|T\right)}=P_{\left(V_{1},V_{2}|T\right)}$.

The Blackwell order $\kappa_{1}⊑\kappa_{2}$ is a preorder of channels, as shown in Equation (5) [14]. It highlights that a channel equivalent to $\kappa_{1}$ can be obtained by garbling the output of $\kappa_{2}$ (a chaining of channels as seen in Equation (5)). Therefore, there exists a decision strategy based on $\kappa_{2}$ for any reward function that performs at least as well as all strategies based on $\kappa_{1}$ [12].

(5) $$\kappa_{1}⊑\kappa_{2}⟺\kappa_{1}=\kappa_{2}\cdot \lambda \;\mathrm{for}\mathrm{some}\mathrm{channel}\lambda$$

Bertschinger and Rauh [12] showed that the Blackwell order does not define a lattice in general since it does not provide a unique meet and joint element beyond binary inputs. However, binary input channels provide a special case for which the Blackwell order is equivalent to the *zonotope order* and defines a lattice. We use the notation $\kappa^{t}$ to indicate that a channel has a binary input (|T|=2) or corresponds to the one-vs-rest encoding for one state *t* if |T|>2. In this case, the row stochastic matrix representing a channel contains a set of vectors ${\vec{v}}_{s}$ as shown in Equation (6). A zonotope $Z_{\kappa^{t}}$ (Equation (6b)) corresponds to “the image of the unit cube [*…*] under the linear map corresponding to [$\kappa^{t}$]” [12] (p. 2), and the resulting zonotope order $\kappa_{1}^{t}⊑\kappa_{2}^{t}\Leftrightarrow Z_{\kappa_{1}^{t}}\subseteq Z_{\kappa_{2}^{t}}$ is a preorder that is identical to the Blackwell order in the special case of binary input channels [12] as visualized in Figure 3. In the resulting lattice, the joint of two channels can be obtained as the convex hull $Z_{\kappa_{1}^{t}⊔\kappa_{2}^{t}}$ of the zonotopes $Z_{\kappa_{1}^{t}}$ and $Z_{\kappa_{2}^{t}}$, and the meet element $Z_{\kappa_{1}^{t}⊓\kappa_{2}^{t}}$ corresponds to their intersection.

(6a) $$\begin{array}{c} \kappa_{i}^{t}=\left[\begin{array}{cccc} p(S_{i}=s_{1}|T=t)\; & \;p(S_{i}=s_{2}|T=t)\; & \;\ldots \; & \;p(S_{i}=s_{n}|T=t)\\ p(S_{i}=s_{1}|T\neq t)\; & \;p(S_{i}=s_{2}|T\neq t)\; & \;\ldots \; & \;p(S_{i}=s_{n}|T\neq t) \end{array}\right] \end{array}$$

(6b) $$\begin{array}{c} Z_{\kappa_{i}^{t}}=\left\{\sum_{s\in S_{i}}x_{s}\cdot {\vec{v}}_{s}|0\leq x_{s}\leq 1\right\}\;\mathrm{where}\;{\vec{v}}_{s}=\left(\begin{array}{c} p(S_{i}=s|T=t)\\ p(S_{i}=s|T\neq t) \end{array}\right) \end{array}$$

### 2.5. Receiver Operating Characteristic Curves

**Figure 4:**
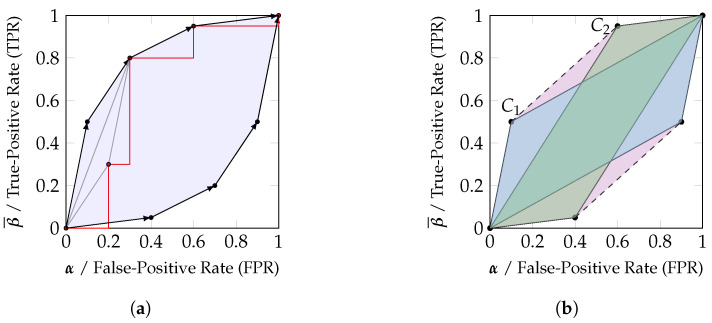
Relating zonotopes and their convex hull to achievable decision regions. (**a**) A ROC curve (red) can be used to estimate the parameters of a channel, and the randomized combination of thresholds (Equation (7)) corresponds to an interpolation in the visualization (gray). The reachable decision region when utilizing all thresholds can be constructed using a likelihood ratio test, which corresponds to reordering the vectors by decreasing slope (in this case, swapping the first two steps) and taking the convex hull of reachable points. This reachable decision region is the zonotope of the channel. (**b**) The convex hull of any set of zonotopes is reachable by their randomized combination. Given two classifiers $C_{1}$ (blue) and $C_{2}$ (green), there always exists a randomized combination that can reach any position in their convex hull (purple).

While any classification system can be represented as channel, this section focuses on binary decision problems or the one-vs-rest encoding of others ($T^{t}=t\Leftrightarrow T=t$). The binary label $t\in T^{t}$ is used to obtain a sample $s\in S_{i}$, which is processed by a classification system *C* to its output o∈O with o=C(s), and applying a decision strategy *d* shall result in an approximation of the label $\hat{t}\in T^{t}$ with $\hat{t}=d\left(o\right)$. This forms the Markov chain: $T^{t}\to S_{i}\to O\to {\hat{T}}^{t}$. A common method of analyzing binary decision/classification systems is the Receiver Operating Characteristic (ROC). A ROC plot typically represents a classifier *C* with a continuous, discrete or categorical output range (by assigning distinct arbitrary values to each category) for a binary decision problem by a curve in a True-Positive Rate (TPR)/False-Positive Rate (FPR) diagram for varying decision thresholds $\tau_{x}$ with the decision rule for a sample *s* being $C\left(s\right)\leq \tau_{x}\Leftrightarrow \mathrm{False}$ [15]. The resulting points are typically connected using a step function, as shown in red in Figure 4a. As a result of using a single decision threshold, the points of the ROC curve monotonically increase from (0,0) to (1,1); however, they are in general neither concave nor convex [16].

We want to highlight the distinction between a ROC curve and the achievable performance pairs (TPR, FPR) based on the classifier. Any performance pair within the convex hull of the obtained points for constructing the ROC curve can be achieved since the decision strategy of Equation (7) results in an interpolation of the points corresponding to $\tau_{1}\leq \tau_{2}$ with the parameter 0≤h≤1 in the TPR/FPR diagram. Therefore, while a ROC curve *is not* convex in general, the achievable performance region *is* convex in general.

(7) $$\begin{array}{cc} C\left(s\right)\leq \tau_{1} & \Rightarrow \mathrm{False},\\ \tau_{1}<C\left(s\right)\leq \tau_{2} & \Rightarrow \mathrm{Bernoulli}(h),\\ \tau_{2}<C\left(s\right) & \Rightarrow \mathrm{True}. \end{array}$$

When utilizing the set of all available thresholds on the classification output, we can identify the reachable decision regions within the TPR/FPR diagram using the likelihood ratio test, which is well known to be optimal for binary decision problems: Neyman–Pearson theory [17] states that the likelihood ratio test (Equation (8)) provides the minimal type II error (minimal β, maximal TPR $=\bar{\beta }=1-\beta$) for a bounded type I error (FPR, α).

(8) $$\begin{array}{cc} \frac{P(S_{i}=s|T=t)}{P(S_{i}=s|T\neq t)}<\tau & \Rightarrow \mathrm{False},\\ \frac{P(S_{i}=s|T=t)}{P(S_{i}=s|T\neq t)}=\tau & \Rightarrow \mathrm{Bernoulli}(h),\\ \frac{P(S_{i}=s|T=t)}{P(S_{i}=s|T\neq t)}>\tau & \Rightarrow \mathrm{True}. \end{array}$$

Notice that the decision criterion is determined by the slope of each vector in the row stochastic matrix that represents the binary input channel (Equation (6a)). This effective reordering of vectors based on their slope when varying the parameters τ and *h* results in the upper half of the zonotope discussed in Section 2.4 and as visualized in Figure 4a. The lower half of the zonotope is obtained from negating the outcome of the likelihood ratio test. Therefore, the zonotope representation of a channel corresponds to the achievable performance region in a TPR/FPR diagram of a classifier at binary decision problems. When reconsidering Figure 3, the channels $\kappa_{1}^{t}=T^{t}\to O_{1}$ and $\kappa_{2}^{t}=T^{t}\to O_{2}$ may correspond to two classifiers $C_{1}$ and $C_{2}$ whose channel parameters have been estimated from a ROC curve, and the achievable performance regions correspond to the zonotopes in a TPR/FPR diagram. Since the likelihood ratio test is optimal for binary decision problems, there cannot exist a decision strategy that would achieve a performance outside the zonotope. At the same time, the likelihood ratio test can be randomized to reach any desired position within the zonotope.

Finally, notice that the convex hull of any two classification systems is reachable by their randomized combination. We can view each classifier as an observation from a channel $\kappa_{1}^{t}$/$\kappa_{2}^{t}$ about $T^{t}$ and know that there always exists a garbling λ of the joint channel $\kappa_{12}^{t}$ to obtain their convex hull $\kappa_{1}^{t}⊔\kappa_{2}^{t}=\kappa_{12}^{t}\lambda$. Using a likelihood ratio test on $\kappa_{1}^{t}⊔\kappa_{2}^{t}$, any position within the convex hull is reachable as a randomized combination of both classifiers. This has been visualized in Figure 4b. Due to this reason, we will say in Section 3.1 that the convex hull should be fully attributed to the marginal channels $\kappa_{1}^{t}$ and $\kappa_{2}^{t}$.

### 2.6. Lattice Valuations

This section summarizes the properties of consistent lattice valuations based on Knuth [13]. The *quantification* of a lattice (L,≼) or (L,⋏,⋎) with α⋏β=α⇔α≼β for elements of the set α,β∈L is a function q:L→R, which assigns reals to each element. A quantification is called a *valuation* if any two elements maintain an ordering relation: α≼β implies that q(α)≤q(β). A quantification *q* is *consistent* if it satisfies a sum rule (inclusion–exclusion principle): q(α⋎β)=q(α)+q(β)−q(a⋏β). If the bottom element of the lattice (*⊥*) is evaluated to zero q(⊥)=0, then the valuation of the Cartesian product of two lattices q((α;β))=q(α)·q(β) remains consistent with the individual lattices. Finally, a *bi-quantification* can be defined as b(α,β)=q(α⋏β)/q(β). Similar to Knuth [13], we will use the notation q([α;β])≡b(α,β) which can be thought of as quantifying a degree of inclusion for α within β. The distributive lattice then creates an algebra like probability theory for the consistent valuation, as summarized in Equation (9) [13].

(9) $$\begin{array}{ccc} q(\alpha ⋎\beta ) & =q(\alpha )+q(\beta )-q(\alpha ⋏\beta ) & \left(\mathrm{Sum}\mathrm{rule}\right)\\ q([\alpha ⋎\beta ;\gamma ]) & =q([\alpha ;\gamma ])+q([\beta ;\gamma ])-q([\alpha ⋏\beta ;\gamma ]) & \left(\mathrm{Sum}\mathrm{rule}\right)\\ q((\alpha ;\beta )) & =q(\alpha )\cdot q(\beta ) & \left(\mathrm{Direct}\mathrm{product}\mathrm{rule}\right)\\ q(([\alpha ;\beta ];[\tau ;\upsilon ])) & =q([\alpha ;\tau ])\cdot q([\beta ;\upsilon ]) & \left(\mathrm{Direct}\mathrm{product}\mathrm{rule}\right)\\ q([\beta ⋏\gamma ;\alpha ]) & =q([\gamma ;\alpha ⋏\beta ])\cdot q([\beta ;\alpha ]) & \left(\mathrm{Product}\mathrm{rule}\right)\\ q([\gamma ;\alpha ⋏\beta ]) & =\frac{q([\beta ;\alpha ⋏\gamma ])\cdot q([\gamma ;\alpha ])}{q([\beta ;\alpha ])} & \left(\mathrm{Bayes}’\;\mathrm{Theorem}\right) \end{array}$$

## 3. Quantifying Reachable Decision Regions

We start by studying the decomposition of binary decision problems from an interpretational perspective (Section 3.1). This provides the basis for constructing a distributive lattice in Section 3.2 and demonstrating the structure of a consistent valuation function. Section 3.3 highlights that mutual information is such a consistent valuation and extends the concept from binary decision problems to target variables with an arbitrary finite number of states. The resulting definition of shared information for the PID will be discussed as an application in Section 4.1 together with the tracing of information flows in Section 4.2.

We define an equivalence relation (∼) for binary input channels $\kappa^{t}$, which allows for the removal of zero vectors, the permutation of columns (P representing a permutation matrix) and the splitting/merging of columns with identical likelihood ratios (vectors of identical slope, ℓ∈R), as shown in Equation (10). These operations are invertible using garblings and do not affect the underlying zonotope.

(10a) $$\;\kappa^{t}∼\;\left[\kappa^{t}\;\left[\begin{array}{c} 0\\ 0 \end{array}\right]\right];$$

(10b) $$\;\kappa^{t}∼\;\kappa^{t}P;$$

(10c) $$[\left(1+\ell \right){\vec{v}}_{1}\;{\vec{v}}_{2}\ldots ]∼\left[\begin{array}{cccc} {\vec{v}}_{1} & \ell {\vec{v}}_{1} & {\vec{v}}_{2} & \ldots \end{array}\right].$$

Based on this definition, block matrices cancel at an inverted sign (ℓ=−1) if we allow negative columns, as shown in Equation (11), where $M_{1}$ and $M_{2}$ are some 2×n matrix.

(11) $$M_{1}∼\left[\begin{array}{ccc} M_{1} & M_{2} & -M_{2} \end{array}\right]$$

### 3.1. Motivation and Operational Interpretation

The aim of this section is to provide a first intuition based on a visual example for the methodology that will be used in Section 3.2 to construct a distributive lattice of the reachable decision regions and its consistent valuation. We only consider binary variables $T^{t}=\left\{t,\bar{t}\right\}$ or the one-vs-rest encoding of others ($T^{t}=t\Leftrightarrow T=t$).

**Figure 5:**
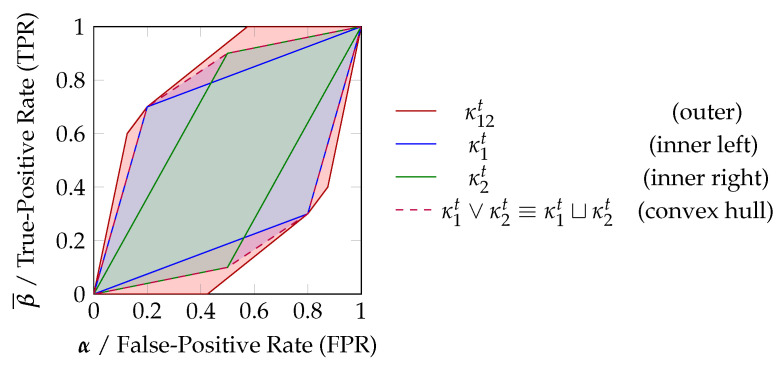
Relating the zonotope representations to TPR/FPR plots. The zonotopes correspond to the regions of a TPR/FPR plot that are reachable by some decision strategy. Regions outside of the zonotopes are known to be unreachable since the likelihood ratio test is optimal for binary decision problems. The convex hull of both zonotopes $\kappa_{1}^{t}\vee \kappa_{2}^{t}$ is the (unique) lower bound of any joint distribution under the Blackwell order.

**Figure 6:**
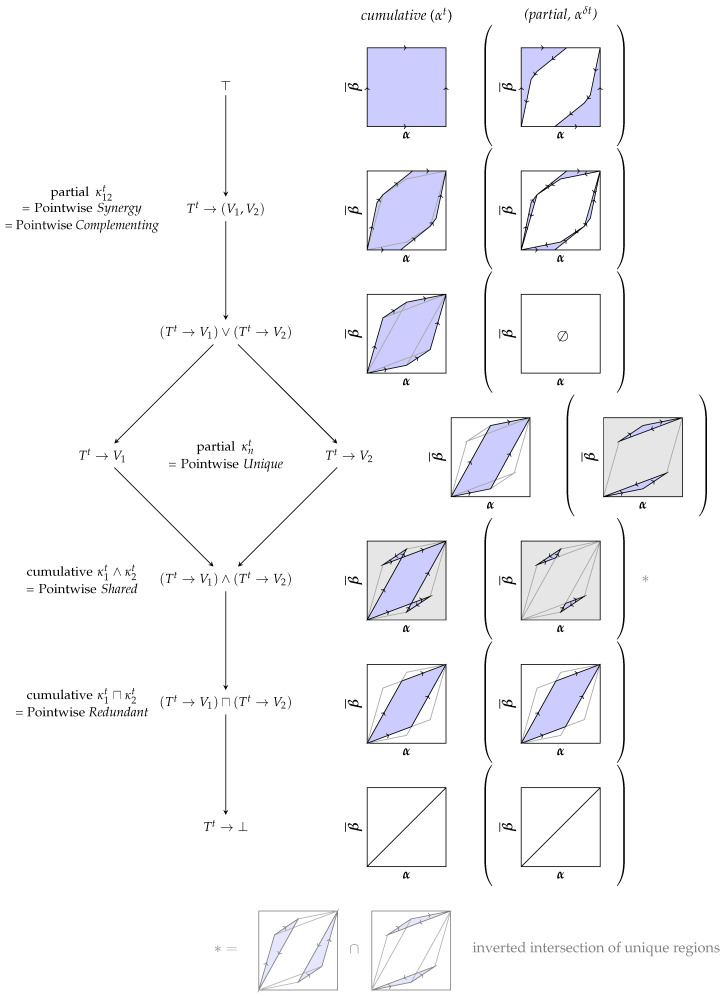
Decomposing the achievable decision regions for binary decision problems from an operational perspective. Each node is visualized by its cumulative and partial decision region. The partial decision region is shown within round brackets. The cumulative region corresponds to the matrix concatenation of the partial regions in its down-set under the defined equivalence relation. Three key elements are highlighted using a grey background.

In the used example, the desired variable can be observed indirectly using the two variables $V_{1}$ and $V_{2}$. The visible variables are considered to be the output of the channels $T^{t}\overset{\kappa_{1}^{t}}{\to }V_{1}$, $T^{t}\overset{\kappa_{2}^{t}}{\to }V_{2}$ and $T^{t}\overset{\kappa_{12}^{t}}{\to }\left(V_{1},V_{2}\right)$ and correspond to the zonotopes shown in Figure 5. We consider each reachable decision point (a pair of TPR and FPR) to represent a different notion of uncertainty about the state of the target variable. We want to attribute the reachable decision regions to each channel for constructing a lattice, as shown in Figure 6, with the following operational interpretation:

- *Synergy:* Corresponds to the partial contribution of $\kappa_{12}^{t}=T^{t}\to \left(V_{1},V_{2}\right)$ and represents the decision region which is only accessible due to the (in-)dependence of both variables.
- *Joint:* The joint element $\kappa_{1}^{t}\vee \kappa_{2}^{t}=\left(T^{t}\to V_{1}\right)\vee \left(T^{t}\to V_{2}\right)$ corresponds to the joint under the Blackwell order and represents the decision region which is always accessible if the marginal distributions $\left(V_{1},T^{t}\right)$ and $\left(V_{2},T^{t}\right)$ can be obtained. Therefore, we say that its information shall be fully attributed to $V_{1}$ and $V_{2}$ such that is has no partial contribution. For binary target variables, this definition is equivalent to the notion of union information by Bertschinger et al. [10] and Griffith and Koch [11]. However, we extend the analysis beyond binary target variables with a different approach in Section 3.3.
- *Unique:* Corresponds to the partial contribution of $\kappa_{1}^{t}=T^{t}\to V_{1}$ or $\kappa_{2}^{t}=T^{t}\to V_{2}$ and represents the decision region that is lost when losing the variable. It only depends on their marginal distributions $\left(V_{1},T^{t}\right)$ and $\left(V_{2},T^{t}\right)$.
- *Shared:* Corresponds to the cumulative contribution of $\kappa_{1}^{t}\wedge \kappa_{2}^{t}=\left(T^{t}\to V_{1}\right)\wedge \left(T^{t}\to V_{2}\right)$ and represents the decision region which is lost when losing either $V_{1}$ or $V_{2}$. Since it only depends on the marginal distributions, we interpret it as being part of both variables. The shared decision region can be split in two components: the decision region that is part of both individual variables and the component that is part of the convex hull but neither individual one. The latter component only exists if both variables provide unique information.
- *Redundant:* The largest decision region $\kappa_{1}^{t}⊓\kappa_{2}^{t}=\left(T^{t}\to V_{1}\right)⊓\left(T^{t}\to V_{2}\right)$ which can be accessed from both $V_{1}$ and $V_{2}$. It corresponds to the meet under the Blackwell order and the part of shared information that can be represented by some random variable (pointwise extractable component of shared information). The redundant and shared regions are equal unless both variables provide some unique information.

Due to the invariance of re-ordering columns under the defined equivalence relation, $\kappa^{t}$ represents a set of likelihood vectors. All cumulative and partial decision regions of Figure 6 can be constructed using a convex hull operator (joint) and matrix concatenations under the defined equivalence relation (∼). For example, the shared decision region (meet) can be expressed through an inclusion–exclusion principle with the joint operator $\kappa_{1}^{t}\wedge \kappa_{2}^{t}∼\left[\begin{array}{ccc} \kappa_{1}^{t}\; & \;\kappa_{2}^{t}\; & \;-\kappa_{1}^{t}\vee \kappa_{2}^{t} \end{array}\right]$. This operator is not closed on channels since it introduces negative likelihood vectors. Therefore, we distinguish the notation between channels ($\kappa^{t}$) and atoms ($\alpha^{t}$). These matrices $\alpha^{t}$ sum to one similar to channels but may contain negative columns. Their partial contributions $\alpha^{\delta t}$ sum to zero.

- The unique contribution of $V_{2}$:                          $\alpha^{\delta t}∼\left[\begin{array}{cc} (\kappa_{1}^{t}\vee \kappa_{2}^{t})\; & \;-\kappa_{1}^{t} \end{array}\right]$
- The shared cumulative region of $V_{1}$ and $V_{2}$:   $\beta^{t}\;∼\left[\begin{array}{ccc} \;\kappa_{1}^{t}\; & \;\kappa_{2}^{t}\; & \;-(\kappa_{1}^{t}\vee \kappa_{2}^{t}) \end{array}\right]∼\kappa_{1}^{t}\wedge \kappa_{2}^{t}$
- The shared partial contribution:                        $\beta^{\delta t}∼\left[\begin{array}{cc} \;\beta^{t}\; & \;-(\kappa_{1}^{t}⊓\kappa_{2}^{t}) \end{array}\right]$
- Each cumulative region corresponds to the combination of partial contributions in its down-set. Notice that the partial contribution of the shared region is canceled by a section of each unique contribution due to an opposing sign:
  $$\kappa_{1}^{t}∼\left[\begin{array}{ccc} \;\alpha^{\delta t}\; & \;\beta^{\delta t}\; & \;(\kappa_{1}^{t}⊓\kappa_{2}^{t}) \end{array}\right]$$

In Section 3.3, we demonstrate a valuation function *f* that can quantify all cumulative and partial atoms of this lattice while ensuring their non-negativity and consistency with the defined equivalence relation (∼). We will refer to a more detailed example on the valuation of partial decision regions in Appendix C in the context of the following section.

Why does the decomposition of reachable decision regions as shown in Figure 6 provide a meaningful operational interpretation? Because combining the partial contributions of the up-set for a variable results in the decision region that becomes inaccessible when the variable is lost, while combining the partial contributions of the down-set results in the decision region that is accessible through the variable. For example, losing access to variable $V_{2}$ results in losing access to the decision regions provided uniquely by $V_{2}$ and its synergy with $V_{1}$ (the up-set on the lattice). Additionally, the cumulative component corresponds to the combination of all partial contributions in its down-set since opposing vectors cancel under the defined equivalence relation (∼) such as the shared and unique contributions. Therefore, we define a consistent valuation of this lattice in Section 3.2 by quantifying decision regions based on their spanning vectors and highlight that the expected value for each t∈T corresponds to the definition of mutual information.

Section 3.2 and Section 3.3 focus only on defining the meet and joint operators (∧/∨) with their consistent valuation. To obtain the pointwise redundant and synergetic components for a PID, we can later add the corresponding channels when constructing the pointwise lattices $V=\{V_{1},V_{2},\left(V_{1},V_{2}\right),V_{1}⊓V_{2}\}$ with the ordering of Figure 6 from the meet and joint operators.

### 3.2. Decomposition Lattice and Its Valuation

This section first defines the meet and joint operators (∧, ∨) and then constructs a consistent valuation for the resulting distributive lattice. For constructing a pointwise channel lattice based on the redundancy lattice, we notate the map of functions as shown in Equation (12) and consider the function $k^{t}\left(S_{i}\right)=T^{t}\to S_{i}=\kappa_{i}^{t}$ to obtain the pointwise channel $\kappa_{i}^{t}$ of a source $S_{i}$.

(12) $$\begin{array}{cc} f\langle P\rangle & =\{f(x)|x\in P\},\\ f\langle \langle P\rangle \rangle & =\{f\langle x\rangle |x\in P\},\\ f\langle \langle \langle P\rangle \rangle \rangle & =\{f\langle \langle x\rangle \rangle |x\in P\}. \end{array}$$

The intersections shall correspond to some meet operation and the union to some joint operation on the pointwise channels, as shown in Equation (13), while maintaining the ordering relation of Williams and Beer [5]. This section aims to define suitable meet and joint operations together with a function for their consistent valuation. Each atom $\alpha^{t},\beta^{t}\in B^{t}\left(V\right)$ now represents an expression of channels $\kappa^{t}$ with the operators ∨/∧, as shown in Appendix A. For example, the element $\left\{S_{12},S_{3}\right\}$ is converted to the expression $\left(\kappa_{1}^{t}\vee \kappa_{2}^{t}\right)\wedge \kappa_{3}^{t}$.

(13) $$B^{t}\left(V\right)=⋀\langle \;⋁\langle \langle \;k^{t}\langle \langle \langle A\left(V\right)\rangle \rangle \rangle \;\rangle \rangle \;\rangle .$$

As seen in Section 3.1, we want to define the joint for a set of channels to be equivalent to their convex hull, matching the Blackwell order. This also ensures that the joint operation is closed on channels.

(14) $$\begin{array}{ccc} \;\;\;\;\kappa_{1}^{t}\vee \kappa_{2}^{t} & \equiv \kappa_{1}^{t}⊔\kappa_{2}^{t}\;\;\; & \left(\mathrm{joint}\mathrm{is}\mathrm{closed}\mathrm{on}\mathrm{channels}\right) \end{array}$$

Since opposing vectors cancel under the defined equivalence relation, we can use a notion of the Möbius inverse to define the set of vectors spanning a partial decision region $\alpha^{\delta t}$ for an atom $\alpha^{t}\in B^{t}\left(V\right)$, as shown in Equation (15), written as a recursive block matrix and using the strict down-set of the ordering based on the underlying redundancy lattice.

(15) $$\alpha^{\delta t}\equiv \left[\begin{array}{cc} \alpha^{t} & -\left[\begin{array}{c} \beta^{\delta t}|\beta^{t}\in \dot{↓}\alpha^{t} \end{array}\right] \end{array}\right]$$

The definition of the meet operator (∧) and the extension of the joint operator (∨) from channels to atoms is now obtained from the constraint that the partial contribution for the joint of two incomparable atoms $\left(\alpha^{t},\beta^{t}\in B^{t}\left(V\right),\alpha^{t}\vee \beta^{t}≁\alpha^{t}\mathrm{and}\alpha^{t}\vee \beta^{t}≁\beta^{t}\right)$ shall be zero, as shown in Equation (16).

(16) $$\alpha^{t}\vee \beta^{t}≁\alpha^{t}\mathrm{and}\alpha^{t}\vee \beta^{t}≁\beta^{t}\;\Rightarrow \;{\left(\alpha^{t}\vee \beta^{t}\right)}^{\delta t}\equiv \left[\begin{array}{c} 0\\ 0 \end{array}\right]$$

This creates the desired inclusion–exclusion principle and results in the equivalences of the meet for two and three atoms, as shown in Equation (17). Their resulting partial channels ($\alpha^{\delta t}$) correspond to the set of vectors spanning the desired unique and shared decision regions of Figure 6.

(17a) $$\begin{array}{cc} \alpha^{t}\wedge \beta^{t}\; & ∼\left[\begin{array}{ccc} \alpha^{t} & \beta^{t} & -\alpha^{t}\vee \beta^{t} \end{array}\right] \end{array}$$

(17b) $$\begin{array}{cc} \alpha^{t}\wedge \left(\beta^{t}\wedge \gamma^{t}\right) & ∼\left[\begin{array}{ccccccc} \alpha^{t} & \beta^{t} & \gamma^{t} & -\alpha^{t}\vee \beta^{t} & -\alpha^{t}\vee \gamma^{t} & -\beta^{t}\vee \gamma^{t} & \alpha^{t}\vee \beta^{t}\vee \gamma^{t} \end{array}\right] \end{array}$$

From their construction, the meet and joint operators provide a distributive lattice for a set of channels under the defined equivalence relation as shown in Appendix B by satisfying idempotency, commutativity, associativity, absorption and distributivity. This can be used to define a corresponding ordering relation (Equation (18)).

(18) $$\alpha^{t}⪯\beta^{t}\equiv \alpha^{t}\wedge \beta^{t}∼\alpha^{t}\Leftrightarrow \alpha^{t}\vee \beta^{t}∼\beta^{t}$$

To obtain a consistent valuation of this lattice, we consider a function $f(\alpha^{t})$, as shown in Equation (19). First, this function has to be invariant under the defined equivalence relation, and second, it has to match the ordering of the constructed lattice.

(19) $$f\left(\alpha^{t}\right)=\sum_{\vec{v}\in \alpha^{t}}r\left(\vec{v}\right)\;\mathrm{where}r\mathrm{is}\mathrm{convex}\mathrm{and}\mathrm{satisfies}\;r\left(\ell \vec{v}\right)=\ell r\left(\vec{v}\right)\;\mathrm{and}\;r\left(\left[\begin{array}{c} \ell \\ \ell \end{array}\right]\right)=0$$

The function *f* shall apply a (convex) function $r(\vec{v})$ to each vector of the matrix of an atom $\vec{v}\in \alpha^{t}$. The function is invariant under the equivalence relation (∼, Equation (10)):

- Zero vectors do not affect the quantification: $r(\left[\begin{array}{c} 0\\ 0 \end{array}\right])=0$ 
- The structure of *f* ensures invariance under reordering columns: $f\left(\kappa^{t}\right)=f\left(\kappa^{t}P\right)$ 
- The property $r\left(\ell \vec{v}\right)=\ell r\left(\vec{v}\right)$ with ℓ∈R ensures invariance under splitting/merging columns of identical likelihood ratios:
  $$f\left(\left[\left(1+\ell \right){\vec{v}}_{1}\right]\right)=\left(1+\ell \right)r\left({\vec{v}}_{1}\right)=r\left({\vec{v}}_{1}\right)+\ell r\left({\vec{v}}_{1}\right)=f\left(\left[{\vec{v}}_{1}\;\;\;\;\ell {\vec{v}}_{1}\right]\right)$$

The function *f* is a consistent valuation of the ordering relation (⪯, Equation (18)) from the constructed lattice:

- The convexity of *r* ensures that the quantification $f(\alpha^{t})$ is a valuation as shown in Appendix C: $\beta^{t}⪯\alpha^{t}\Rightarrow f\left(\beta^{t}\right)\leq f\left(\alpha^{t}\right)$ 
- The function *f* provides a sum-rule: $f\left(\alpha^{t}\wedge \beta^{t}\right)=f\left(\left[\begin{array}{ccc} \alpha^{t} & \;\beta^{t} & \;-\alpha^{t}\vee \beta^{t} \end{array}\right]\right)=f\left(\alpha^{t}\right)+f\left(\beta^{t}\right)-f\left(\alpha^{t}\vee \beta^{t}\right)$ 
- The function *f* quantifies the bottom element correctly: $f\left(⊥\right)=r\left(\left[\begin{array}{c} \ell \\ \ell \end{array}\right]\right)=0$

A parameterized function that forms a consistent lattice valuation with 0≤p≤1 and that will be used in Section 3.3 is shown in Equation (20) (the convexity of $r_{p}$ is shown in Appendix D).

(20a) $$\begin{array}{cc} f_{p}\left(\alpha^{t}\right) & =\sum_{\vec{v}\in \alpha^{t}}r_{p}\left(\vec{v}\right) \end{array}$$

(20b) $$\begin{array}{cc} r_{p}\left(\vec{v}\right) & =r_{p}\left(\left[\begin{array}{c} x\\ y \end{array}\right]\right)=x\log\left(\frac{x}{px+(1-p)y}\right) \end{array}$$

This section demonstrated the construction of a distributive lattice and its consistent valuation, resulting in an algebra as shown in Equation (9).

### 3.3. Decomposing Mutual Information

This section demonstrates that mutual information is the expected value of a consistent valuation for the constructed pointwise lattices and discusses the resulting algebra. To show this, we define the parameter *p* and pointwise channel $\kappa_{i}^{t}$ for the consistent valuation (Equation (20)) using a one-vs-rest encoding (Equation (21)).

(21) $$\begin{array}{ccc} p & =P_{\left(T=t\right)} & \left(\mathrm{parameter}\right)\\ \kappa_{i}^{t} & =\left[\begin{array}{c} P_{\left(S_{i}|T=t\right)}\\ P_{\left(S_{i}|T\neq t\right)} \end{array}\right]=\left[\begin{array}{cccc} x_{1} & x_{2} & \ldots & x_{m}\\ y_{1} & y_{2} & \ldots & y_{m} \end{array}\right] & \left(\mathrm{binary}\mathrm{input}\mathrm{channel}\right) \end{array}$$

The expected value of the resulting valuation in Equation (20) is equivalent to the definition of mutual information, as shown in Equation (22). Therefore, we can interpret mutual information as being the expected value of quantifying the reachable decision regions for each state of the target variable that represent a concept of pointwise uncertainty.

(22a) $$\begin{array}{cc} I(T;S_{i}) & =\sum_{s\in S_{i}}\sum_{t\in T}P_{\left(S_{i},T\right)}\left(s,t\right)\log\left(\frac{P_{\left(S_{i},T\right)}\left(s,t\right)}{P_{S_{i}}\left(s\right)P_{T}\left(t\right)}\right) \end{array}$$

(22b) $$\begin{array}{cc} \; & =E_{T}\left[\sum_{s\in S_{i}}\underset{x_{j}}{\underset{︸}{P_{\left(S_{i}|T=t\right)}\left(s\right)}}\log\left(\frac{\overset{x_{j}}{\overset{︷}{P_{\left(S_{i}|T=t\right)}\left(s\right)}}}{\underset{p}{\underset{︸}{P_{\left(T=t\right)}}}\underset{x_{j}}{\underset{︸}{P_{\left(S_{i}|T=t\right)}\left(s\right)}}+\underset{1-p}{\underset{︸}{\left(1-P_{\left(T=t\right)}\right)}}\underset{y_{j}}{\underset{︸}{P_{\left(S_{i}|T\neq t\right)}\left(s\right)}}}\right)\right] \end{array}$$

The expected value for a set of consistent lattice valuations corresponds to a weighted sum such that the resulting lattice remains consistent. Therefore, we can combine the pointwise lattices to extend the definition of mutual information for meet and joint elements, which we will think of as intersections and unions. Let α represent an expression of sources with the operators ∨ and ∧. Then, we can obtain its valuation from the pointwise lattices using the function ${\hat{I}}_{T}$, as shown in Equation (23). Notice that we do not define the operators for random variables but only use the notation for selecting the corresponding element on the underlying pointwise lattices. For example, we write $\alpha =\left(S_{12}\wedge S_{3}\right)\vee S_{4}$ to refer to the pointwise atom $\alpha^{t}=\left(\kappa_{12}^{t}\wedge \kappa_{3}^{t}\right)\vee \kappa_{4}^{t}$ on each pointwise lattice.

The special case of atoms that consist of a single source corresponds by construction to the definition of mutual information. However, we propose normalizing the measure, as shown in Equation (23), to capture a degree of inclusion between zero and one. This is possible for discrete variables and will lead to an easier intuition for the later definition of bi-valuations and product spaces by ensuring the same output range for these measures. As a possible interpretation for the special role of the target variable, we like to think of *T* as the considered origin of information within the system, which then propagates through channels to other variables.

(23) $$\begin{array}{ccccc} {\hat{I}}_{T}\left(\alpha \right) & \equiv \frac{E_{T}\left[f_{P_{T}\left(t\right)}\left(\alpha^{t}\right)\right]}{E_{T}\left[f_{P_{T}\left(t\right)}\left(⊤\right)\right]} & \; & =\frac{E_{T}\left[f_{P_{T}\left(t\right)}\left(\alpha^{t}\right)\right]}{H(T)} & \\ {\hat{I}}_{T}\left(T\right) & =1 & \; & =\frac{H(T)}{H(T)}\\ {\hat{I}}_{T}\left(S_{i}\right) & ={\hat{I}}_{T}\left(T\wedge S_{i}\right) & \; & =\frac{I(T;S_{i})}{H(T)} \end{array}$$

We obtain the following algebra with the bi-valuation ${\hat{I}}_{T}\left(\left[\alpha ;\;\beta \right]\right)$ that quantifies a degree of inclusion from α within the context of β. We can think of ${\hat{I}}_{T}\left(\left[\alpha ;\;\beta \right]\right)$ as asking how much of the information from β about *T* is shared with α.

(24a) $$\begin{array}{ccc} {\hat{I}}_{T}\left(\alpha \vee \beta \right) & ={\hat{I}}_{T}\left(\alpha \right)+{\hat{I}}_{T}\left(\beta \right)-{\hat{I}}_{T}\left(\alpha \wedge \beta \right) & \left(\mathrm{Sum}\mathrm{rule}\right) \end{array}$$

(24b) $$\begin{array}{ccc} {\hat{I}}_{T}\left(\left[\alpha ;\;\beta \right]\right) & \equiv \frac{{\hat{I}}_{T}\left(\alpha \wedge \beta \right)}{{\hat{I}}_{T}\left(\beta \right)} & \left(\mathrm{Bi}\text{-}\mathrm{Valuation}\right) \end{array}$$

(24c) $$\begin{array}{ccc} {\hat{I}}_{T}\left(\left[\alpha \vee \beta ;\;\gamma \right]\right) & ={\hat{I}}_{T}\left(\left[\alpha ;\;\gamma \right]\right)+{\hat{I}}_{T}\left(\left[\beta ;\;\gamma \right]\right)-{\hat{I}}_{T}\left(\left[\alpha \wedge \beta ;\;\gamma \right]\right) & \left(\mathrm{Conditioned}\mathrm{sum}\mathrm{rule}\right) \end{array}$$

(24d) $$\begin{array}{ccc} {\hat{I}}_{T}\left(\left[\beta \wedge \gamma ;\;\alpha \right]\right) & ={\hat{I}}_{T}\left(\left[\gamma ;\;\alpha \wedge \beta \right]\right)\cdot {\hat{I}}_{T}\left(\left[\beta ;\;\alpha \right]\right) & \left(\mathrm{Product}\mathrm{rule}\right) \end{array}$$

(24e) $$\begin{array}{ccc} {\hat{I}}_{T}\left(\left[\beta ;\;\alpha \wedge \gamma \right]\right) & =\frac{{\hat{I}}_{T}\left(\left[\gamma ;\;\alpha \wedge \beta \right]\right)\cdot {\hat{I}}_{T}\left(\left[\beta ;\;\alpha \right]\right)}{{\hat{I}}_{T}\left(\left[\gamma ;\;\alpha \right]\right)} & \left(\mathrm{Bayes}’\;\mathrm{Theorem}\right) \end{array}$$

Since the definitions satisfy an inclusion–exclusion principle, we obtain the interpretation of classical measures as proposed by Williams and Beer [5]: conditional mutual information $I(T;V_{1}|V_{2})$ measures the unique contribution of $V_{1}$ plus its synergy with $V_{2}$, and interaction information $I(T;V_{1};V_{2})$ measures the difference between synergy and shared information, which explains its possible negativity.

As highlighted by Knuth [13], the lattice product (the Cartesian product with ordering (α;β)⪯(τ;υ)⇔α⪯τandβ⪯υ) can be valuated using a product rule to maintain consistency with the ordering of the individual lattices. This creates an opportunity to define information product spaces for multiple reference variables. Since we normalized the measures, the valuation of the product space will also be normalized to the range from zero to one. The subscript notation $T_{1}\times T_{2}$ shall indicate the product of the lattice constructed for $T_{1}$ with the product of the lattice constructed for $T_{2}$.

(25a) $$\begin{array}{ccc} {\hat{I}}_{\left(T_{1}\times T_{2}\right)}\left(\left(\alpha ;\;\beta \right)\right) & ={\hat{I}}_{T_{1}}\left(\alpha \right)\cdot {\hat{I}}_{T_{2}}\left(\beta \right) & \left(\mathrm{Valuation}\mathrm{Product}\mathrm{rule}\right) \end{array}$$

(25b) $$\begin{array}{ccc} {\hat{I}}_{\left(T_{1}\times T_{2}\right)}\left(\left(\left[\alpha ;\;\tau \right];\;\left[\beta ;\;\upsilon \right]\right)\right) & ={\hat{I}}_{T_{1}}\left(\left[\alpha ;\;\beta \right]\right)\cdot {\hat{I}}_{T_{2}}\left(\left[\tau ;\upsilon \right]\right) & \left(\mathrm{Bi}\text{-}\mathrm{Valuation}\mathrm{Product}\mathrm{rule}\right) \end{array}$$

The lattice product is distributive over the joint for disjoint elements [13], which leads to the equivalence in Equation (26). Unfortunately, it appears that only the bottom element is disjoint with other atoms in the constructed lattice.

(26) $$\forall t:\alpha^{t}\wedge \beta^{t}∼⊥\;\Rightarrow \;{\hat{I}}_{\left(T_{1}\times T_{2}\right)}\left(\left(\alpha \vee \beta ;\;\tau \right)\right)={\hat{I}}_{\left(T_{1}\times T_{2}\right)}\left(\left(\alpha ;\;\tau \right)\vee \left(\beta ;\;\tau \right)\right)$$

Finally, we would like to provide an intuition for this approach based on possible operational scenarios:

1. Consider having characterized four radio links and obtained the conditional distributions $P_{V_{1}|T}$, $P_{(V_{2},V_{3})|T}$ and $P_{V_{4}|T}$. We are interested in their joint channel capacity; however, lack the required joint distribution. In this case, we can use their joint $\sup_{P_{T}\left(t\right)}{\hat{I}}_{T}\left(S_{1}\vee S_{23}\vee S_{4}\right)$ to obtain a (pointwise) lower bound on their joint channel capacity.
2. Consider having two datasets $\left\{T_{1},V_{1},V_{2},V_{3}\right\}$ and $\left\{T_{2},V_{2},V_{3},V_{4}\right\}$ that provide different types of labels ($T_{x}$) and associated features ($V_{y}$), where some events were recorded in both datasets. In such cases, one may choose to study the cases $T_{1}\to \left(V_{1},V_{2},V_{3}\right)$, $T_{2}\to \left(V_{2},V_{3},V_{4}\right)$ and $\left(T_{1},T_{2}\right)\to \left(V_{1},V_{2},V_{3},V_{4}\right)$ for events appearing in both datasets, which could then be combined into a product lattice ${\hat{I}}_{\left(T_{1}\times T_{2}\times \left(T_{1},T_{2}\right)\right)}$.

## 4. Applications

This section focuses on applications of the obtained measure from Section 3.3. We first apply the meet operator to the redundancy lattice for constructing a PID. Since an atom of the redundancy lattice α∈A(V) corresponds to a set of sources for which the shared information shall be measured, we use the notation ⋀α to obtain an expression for the function ${\hat{I}}_{T}$. Section 4.2 additionally utilizes the properties of a Markov chain to demonstrate how the flow of partial information can be traced through system models.

### 4.1. Partial Information Decomposition

Based on Section 3.3, we can define a measure of shared information $\hat{I}\left(\alpha ;T\right)$ for the elements of the redundancy lattice α∈A(V) in the framework of Williams and Beer [5], as shown in Equation (27). The measure satisfies the three axioms of Williams and Beer [5] (commutativity from the equivalence relation and structure of $f_{p}$, monotonicity from being a lattice valuation and self-redundancy from removing the normalization), and the decomposition is non-negative since the joint channel $\kappa_{12}^{t}$ is superior to the joint of two channels $\kappa_{1}^{t}\vee \kappa_{2}^{t}$ for all t∈T. The partial contribution ${\hat{I}}^{\delta }\left(\alpha ;T\right)$ corresponds to the expected value of the quantified partial decision regions $\alpha^{\delta t}$.

This provides the interpretation of Section 3.1, where combining the partial contributions of the up-set corresponds to the expected value of quantifying the decision regions that are lost when losing the variable, while combining the partial contributions of the down-set corresponds to the expected value of quantifying the accessible decision region from this variable. Additionally, we obtain a pointwise version of the property by Bertschinger et al. [10]: if a variable provides unique information, then there is a way to utilize this information for a reward function to some target variable state. Finally, it can be seen that taking the minimal quantification of the different decision regions as done by Williams and Beer [5] leads to a lack in distinguishing distinct reachable decision regions or, as phrased in the literature: a lack of distinguishing “the *same* information and the *same amount* of information” [6,7,8,9].

(27a) $$\begin{array}{cc} \forall \alpha \in A\left(V\right),\;\hat{I}\left(\alpha ;T\right) & ={\hat{I}}_{T}\left(⋀\alpha \right)\cdot H\left(T\right), \end{array}$$

(27b) $$\begin{array}{cc} {\hat{I}}^{\delta }\left(\alpha ;T\right) & =\hat{I}\left(\alpha ;T\right)-\sum_{\beta \in \dot{↓}\alpha }{\hat{I}}^{\delta }\left(\beta ;T\right)=E_{T}\left[f_{P_{T}\left(t\right)}\left(\alpha^{\delta t}\right)\right] \end{array}$$

An identical definition of $\hat{I}\left(\alpha ;T\right)$ can be obtained only based on the Blackwell order, as shown in Equation (28). Let α∈A(V) be a set of sources and let $T^{t}$ represent a binary target variable ($T^{t}=\left\{t,\bar{t}\right\}$) such that $T^{t}=t\Leftrightarrow T=t$. We can expand the meet operator used in Equation (27a) using the sum-rule and utilize the distributivity for arriving at the joint of two channels, which matches the Blackwell order (Equation (28b)). We write $S_{i}{⊔}_{T^{t}}S_{j}$ to refer to the joint of $S_{i}$ and $S_{j}$ under the Blackwell order with respect to variable $T^{t}$. This results in the recursive definition of $i\left(\alpha ;T^{t}\right)$ that corresponds to the definition of mutual information for a single source (Equation (28a)). This expansion of Equation (27a) is particularly helpful since it eliminates the operators ∧/∨ for a simplified implementation.

(28a) $$\begin{array}{cc} i\left(\left\{S_{i}\right\};T^{t}\right) & =\sum_{s\in S_{i}}P_{\left(S_{i}|T^{t}=t\right)}\left(s\right)\log\left(\frac{P_{\left(S_{i}|T^{t}=t\right)}\left(s\right)}{P_{\left(T^{t}=t\right)}P_{\left(S_{i}|T^{t}=t\right)}\left(s\right)+\left(1-P_{\left(T^{t}=t\right)}\right)P_{\left(S_{i}|T^{t}\neq t\right)}\left(s\right)}\right) \end{array}$$

(28b) $$\begin{array}{cc} i\left(\left\{S_{i}\right\}\cup \beta ;T^{t}\right) & =i\left(\left\{S_{i}\right\};T^{t}\right)+i\left(\beta ;T^{t}\right)-i\left(\left\{S_{i}{⊔}_{T^{t}}S_{j}|S_{j}\in \beta \right\};T^{t}\right) \end{array}$$

(28c) $$\begin{array}{cc} \hat{I}\left(\alpha ;T\right) & =E_{T}\left[i\left(\alpha ,T^{t}\right)\right] \end{array}$$

Our decomposition is equivalent to the measures of Bertschinger et al. [10], Griffith and Koch [11] and Williams and Beer [5] in two special cases:

- For a binary target variable $T=\{t,\bar{t}\}$ with two observable variables $V_{1}$ and $V_{2}$, our approach is identical to Bertschinger et al. [10] and Griffith and Koch [11] since $\kappa_{1}⊔\kappa_{2}∼\kappa_{1}^{t}\vee \kappa_{2}^{t}∼\kappa_{1}^{\bar{t}}\vee \kappa_{2}^{\bar{t}}$. Beyond binary target variables, the resulting definitions differ due to the pointwise construction (see Appendix E). 
- If from a pointwise perspective ($T^{t}$), some variable is Blackwell superior to the other (not necessarily the same each time), then our method is identical to Williams and Beer [5] since the defined meet operation will equal their minimum $\kappa_{1}^{t}⊔\kappa_{2}^{t}∼\kappa_{2}^{t}\Rightarrow f_{p}\left(\kappa_{1}^{t}\right)\leq f_{p}\left(\kappa_{2}^{t}\right)\Rightarrow \min\left(f_{p}\left(\kappa_{1}^{t}\right),f_{p}\left(\kappa_{2}^{t}\right)\right)=f_{p}\left(\kappa_{1}^{t}\wedge \kappa_{2}^{t}\right)=f_{p}\left(\kappa_{1}^{t}\right)$ and equivalently for the function $i(\alpha ,T^{t})$.

A decomposition of typical examples can be found in Appendix E. We also provide an implementation of the PID based on our approach [18].

### 4.2. Information Flow Analysis

Due to the achieved inclusion–exclusion principle, the data processing inequality of mutual information and the achieved non-negativity of partial information for an arbitrary number of variables, it is possible to trace the flow of information through Markov chains. The measure ${\hat{I}}_{T}$ appears suitable for this analysis due to the chaining properties of the underlying pointwise channels that are quantified. The analysis can be applied among others for analyzing communication networks or designing data processing systems.

The flow of information in Markov chains has been studied by Niu and Quinn [19], who considered chaining individual variables $X_{1}\to X_{2}\to \ldots \to X_{n}$ and performed a decomposition on $V=\{X_{1},X_{2},\ldots ,X_{n}\}$. In contrast to this, we consider Markov chains that map sets of random variables from one step to the next. In this case, it is possible to perform an information decomposition at each step of the Markov chain and identify how the partial information components propagate from one set of variables to the next.

Let T→V→Q be a Markov chain with the atoms α∈A(V) and β∈A(Q), through which we trace the flow of partial information from α to β about *T*. We can measure the shared information between both atoms α and β, as shown in Equation (29a), to obtain how much information their cumulative components share ${\hat{J}}^{\cap \to \cap }\left(\alpha \to \beta ;T\right)$. Similar to the PID, we remove the normalization for the self-redundancy axiom. To identify how much of the cumulative information of β is obtained from the partial information of α, we subtract the strict down-set of α on the lattice (A(V),≼) as shown in Equation (29b) to obtain ${\hat{J}}^{\delta \to \cap }\left(\alpha \to \beta ;T\right)$. To compute how much of the partial information of α is shared with the partial contribution of β, we similarly remove the flow from the partial information of α into the strict down-set of β on the lattice (A(Q),≼), as shown in Equation (29c), to obtain ${\hat{J}}^{\delta \to \delta }\left(\alpha \to \beta ;T\right)$. This can be used to trace the origin of information for each atom β∈A(Q) to the previous elements α∈A(V).

The approach is not limited to one step and can be extended for tracing the flow through Markov chains of arbitrary length ${\hat{J}}^{\delta \to \delta \to \delta \;\ldots }\left(\alpha \to \beta \to \gamma \;\ldots ;T\right)$. However, we only trace one step in this demonstration for simplicity.

(29a) $$\begin{array}{cc} {\hat{J}}^{\cap \to \cap }\left(\alpha \to \beta ;T\right) & ={\hat{I}}_{T}\left(⋀\alpha \wedge ⋀\beta \right)\cdot H\left(T\right) \end{array}$$

(29b) $$\begin{array}{cc} {\hat{J}}^{\delta \to \cap }\left(\alpha \to \beta ;T\right) & ={\hat{J}}^{\cap \to \cap }\left(\alpha \to \beta ;T\right)-\sum_{\gamma \in \dot{↓}\alpha }{\hat{J}}^{\delta \to \cap }\left(\gamma \to \beta ;T\right) \end{array}$$

(29c) $$\begin{array}{cc} {\hat{J}}^{\delta \to \delta }\left(\alpha \to \beta ;T\right) & ={\hat{J}}^{\delta \to \cap }\left(\alpha \to \beta ;T\right)-\sum_{\gamma \in \dot{↓}\beta }{\hat{J}}_{T}^{\delta \to \delta }\left(\alpha \to \gamma ;T\right) \end{array}$$

We demonstrate the Information Flow Analysis using a full-adder as a small logic circuit with the input variables $V=\{A,B,C_{\mathrm{in}}\}$ and the output $T=\{S,C_{\mathrm{out}}\}$ as shown in Equation (30). Any ideal implementation of this computation results in the same channel from V to T. Therefore, they create an identical flow of the partial information from V to the partial information of T. However, the specific implementation will determine how (over which intermediate representations and paths) the partial information is transported.

(30) $$\begin{array}{ccc} S & =A⊕B⊕C_{\mathrm{in}}\\ \;C_{\mathrm{out}} & =A\cdot B+A\cdot C_{\mathrm{in}}+B\cdot C_{\mathrm{in}} & \\ \; & =\left(A\cdot B\right)+C_{\mathrm{in}}\cdot \left(A⊕B\right)) & \;\;\left(\mathrm{typical}\mathrm{implementation}\right)\\ T & =(S,C_{\mathrm{out}}) \end{array}$$

**Figure 7:**
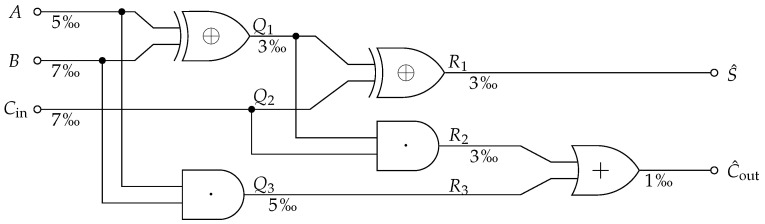
Noisy full-adder example for the Information Flow Analysis demonstration. The probability of a bit-flip is indicated below the wires. If a wire has two labels, the first label corresponds to the wire input and the second label to its output.

To make the example more interesting, we consider the implementation of a noisy full-adder, as shown in Figure 7, which allows for bit-flips on wires. We indicate the probability of a bit-flip below each line and imagine this value correlates to the wire length and proximity to others. Now, changing the implementation or even the layout of the same circuit would have an impact on the overall channel.

To perform the analysis, we first have to define the target variable: What it is that we want to measure information about? In this case, we select the joint distribution of the desired computation output *T* as the target variable and define the noisy computation result to be $\hat{T}=\left\{\hat{S},{\hat{C}}_{\mathrm{out}}\right\}$, as shown in Figure 7. We obtain both variables from their definition by assuming that the input variables V are independently and uniformly distributed and that bit-flips occurred independently. However, it is worth noting that noise dependencies can be modeled in the joint distribution. This fully characterizes the Markov chain shown in Equation (31).

(31) $$T=\left(S,C_{\mathrm{out}}\right)\to T=\left\{S,C_{\mathrm{out}}\right\}\to V=\left\{A,B,C_{\mathrm{in}}\right\}\to Q=\left\{Q_{1},Q_{2},Q_{3}\right\}\to R=\left\{R_{1},R_{2},R_{3}\right\}\to \hat{T}=\left\{\hat{S},{\hat{C}}_{\mathrm{out}}\right\}$$

**Figure 8:**
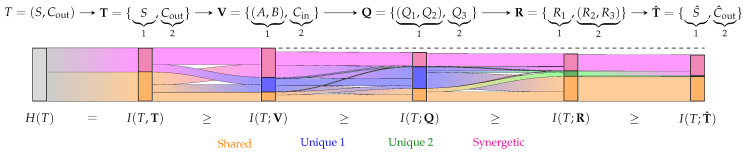
Sankey diagram of the Information Flow Analysis for the noisy full-adder in Figure 7. Each bar corresponds to one stage in the Markov chain, and its height corresponds to this stage’s mutual information with the target *T*. Each bar is decomposed into the information that the considered variables provide shared (orange), unique (blue/green) or synergetic (pink) about the target. If a stage is represented by a single variable or joint distribution, no further decomposition is performed (gray). We trace the information between variables over one step using the sub-chains T→T→T, T→T→V, T→V→Q, T→Q→R and $T\to R\to \hat{T}$ using Equation (29). The resulting flows between each bar visualize how the partial information propagates for one step in the Markov chain. For following the flow of a particular partial component over more than one step in the Sankey diagram, Equation (29) can be extended.

We group two variables at each stage to reduce the number of interactions in the visualization. The resulting information flow of the full-adder is shown as a Sankey diagram in Figure 8. Each bar corresponds to the mutual information of a stage in the Markov chain with the input *T*. The bars’ colors indicate the partial information decomposition of Equation (27). The information flow over one step using Equation (29) is indicated by the width of a line between the partial contributions of two stages. To follow the flow of a particular component over more than one step—for example, to see how the shared information of T propagates to the shared information of $\hat{T}$—the analysis can be performed by tracing multiple steps after extending Equation (29).

The results (Figure 8) show that the decomposition does not attribute unique information to *S* or $C_{\mathrm{out}}$ about their own joint distribution. The reason for this is shown in Equation (32): both variables provide an equivalent channel for each state of their joint distribution and, thus, an equivalent uncertainty about each state of *T*. Phrased differently, both variables provide access to the identical decision regions for each state of their joint distribution and can therefore not provide unique information (no advantage for any reward function to any t∈T). If this result feels counter-intuitive, we would also recommend the discussion of the two-bit-copy problem and identity axiom by Finn [9] (p. 16ff.) and Finn and Lizier [20]. The same effect can also be seen when viewing each variable in V individually (not shown in Figure 8), which causes neither of them to provide unique information on their own about the joint target distribution *T*.

(32) $$\begin{array}{c} \left(T^{\left(0,0\right)}\to C_{{}_{\mathrm{out}}}\right)∼\left(T^{\left(0,0\right)}\to S\right)∼\left[\begin{array}{cc} 1 & 0\\ 3/7 & 4/7 \end{array}\right]∼\left(T^{\left(1,1\right)}\to C_{{}_{\mathrm{out}}}\right)∼\left(T^{\left(1,1\right)}\to S\right)\\ \left(T^{\left(0,1\right)}\to C_{{}_{\mathrm{out}}}\right)∼\left(T^{\left(0,1\right)}\to S\right)∼\left[\begin{array}{cc} 1 & 0\\ 1/5 & 4/5 \end{array}\right]∼\left(T^{\left(1,0\right)}\to C_{{}_{\mathrm{out}}}\right)∼\left(T^{\left(1,0\right)}\to S\right) \end{array}$$

The Information Flow Analysis is particularly useful in practice since it can be performed on an arbitrary resolution of the system model to handle its complexity. For example, a small full-adder can be analyzed on the level of gates and wires represented by channels. However, the full-adder is itself a channel that can be used to analyze an *n*-bit adder on the level of full-adders.

Further applications of the Information Flow Analysis could include the identification of which inputs are most critical for the computational result and where information is being lost. It can also be explored if a notion of robustness in data processing systems could be meaningfully defined based on how much pointwise redundant or shared information of the input V can be traced to its output $\hat{T}$. This might indicate a notion of robustness based on whether or not it is possible to compensate for the unavailability of input sources through a system modification.

Finally, the target variable does not have to be the desired computational outcome as has been done in the demonstration. When thinking about secure multi-party computations, it might be of interest to identify the flow of information from the perspective of some sensitive or private variable (*T*) to understand the impact of disclosing the final computation result. The possible applications of such an analysis are as diverse as those of information theory.

## 5. Discussion

We propose the interpretation that the reachable decision regions correspond to different notions of uncertainty about each state of the target variable and that mutual information corresponds to the expected value of quantifying these decision regions. This allows partial information to represent the expected value of quantifying partial decision regions (Equations (27) and (28)), which can be used to attribute mutual information to the visible variables and their interactions (pointwise redundant/shared/unique/synergetic). Since the proposed quantification results in the consistent valuation of a distributive lattice, it creates a novel algebra for mutual information with possible practical applications (Equations (24) and (25)). Finally, the approach allows for tracing information components through Markov chains (Equation (29)), which can be used to model and study a wide range of scenarios. The presented method is directly applicable to discrete and categorical source variables due to their equivalent construction for the reachable decision regions (zonotopes). However, we recommend that the target variable should be categorical since the measure does not consider a notion of distance between target states (achievable estimation proximity). This would be an interesting direction for future work due to its practical application for introducing semantic meaning to sets of variables. An intuitive example is a target variable with 256 states that is used to represent an 8-bit unsigned integer as the computation result. For this reason, we wonder if it is possible to introduce a notion of distance to the analysis such that the classical definition of mutual information becomes the special case for encoding categorical targets.

A recent work by Kolchinsky [21] removes the assumption that an inclusion–exclusion principle relates the intersection and union of information and demands their extractability. This has the disadvantage that a similar algebra or tracing of information would no longer be possible. We tried to address this point by distinguishing the *pointwise redundant* from the *pointwise shared* element and also obtain no inclusion–exclusion principle for the pointwise redundancy. We focus in this work on the pointwise shared element due to the resulting properties and operational interpretation from the accessibility and losses of reachable decision regions. Moreover, the relation between the used meet and joint operators provides consistent results from performing the decomposition using the meet operator on a redundancy lattice, as done in this work, or a decomposition using the joint operator on a synergy or loss lattice [22].

Further notions of redundancy and synergy can be studied within this framework if they are extractable, meaning they can be represented by some random variable. Depending on the desired interpretation, the representing variable can be constructed for *T* and added to the set of visible variables or can be constructed for each pointwise variable $T^{t}$ and added to the pointwise lattices. We showed an example of the latter in Section 3.1 by adding the pointwise redundant element to the lattice, which we interpret as pointwise extractable components of shared information to quantify the decision regions that can be obtained from each source.

**Figure A3:**
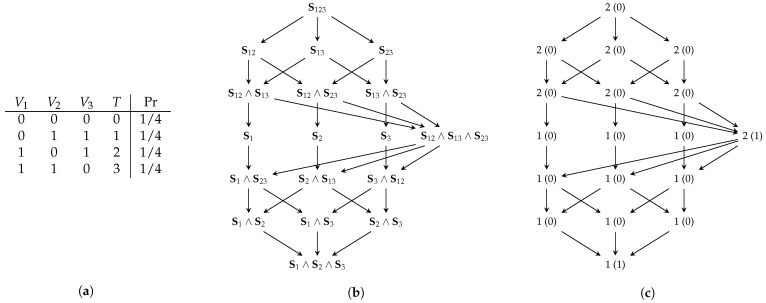
Three-bit even-parity (Tbep) example. The results for ${\hat{I}}_{T}\cdot H\left(T\right)$, $I_{\min}$ and $I^{\pm }$ are identical. (**a**) Distribution. (**b**) Decomposition lattice. (**c**) Cumulative results (partial).

Since our approach satisfies the original axioms of Williams and Beer [5] and results in non-negative partial contributions for an arbitrary number of variables, it cannot satisfy the proposed identity axiom of Harder et al. [8]. This can also be seen by the decomposition examples in Appendix E (Table A2 and Figure A3). We do not consider this a limitation since all four axioms cannot be satisfied without obtaining negative partial information [23], which creates difficulties for interpreting results.

Finally, our approach does not appear to satisfy a target/left chain rule as proposed by Bertschinger et al. [7]. While our approach provides an algebra that can be used to handle multiple target variables, we think that further work on understanding the relations when decomposing with multiple target variables is needed. In particular, it would be helpful for the analysis of complex systems if the flow of already analyzed sub-chains could be reused and their interactions could be predicted.

## 6. Conclusions

We use the approach of Bertschinger et al. [10] and Griffith and Koch [11] to construct a pointwise partial information decomposition that provides non-negative results for an arbitrary number of variables and target states. The measure obtains an algebra from the resulting lattice structure and enables the analysis of complex multivariate systems in practice. To our knowledge, this is the first alternative to the original measure of Williams and Beer [5] that satisfies their three proposed axioms and results in a non-negative decomposition for an arbitrary number of variables.

## Figures and Tables

**Figure A1:**
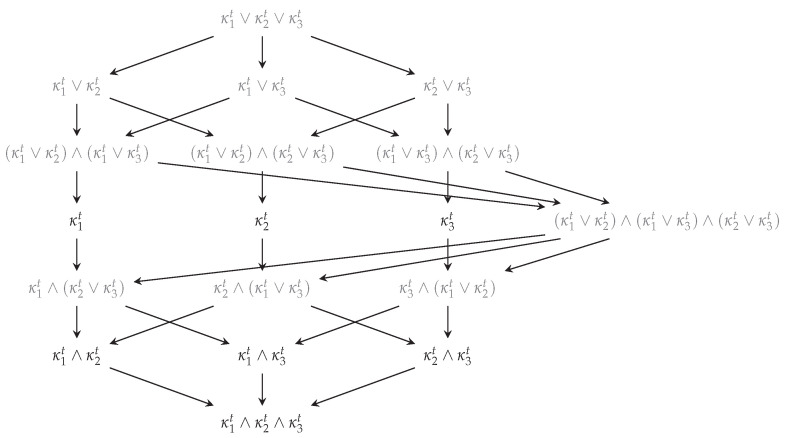
The considered lattice relating the meet and joint operators. The joint of any two incomparable elements ($\alpha^{t},\beta^{t}\in B^{t}\left(V\right),\alpha^{t}\vee \beta^{t}≁\alpha^{t}\mathrm{and}\alpha^{t}\vee \beta^{t}≁\beta^{t}$) shall have no partial contribution to create an inclusion–exclusion principle between the operators and is highlighted using a gray font.

**Figure A2:**
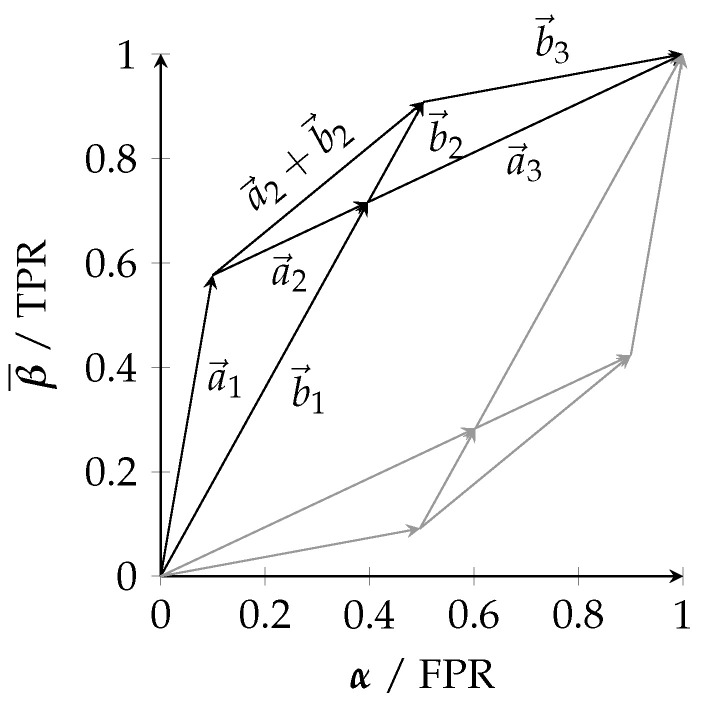
A minimal example to discuss the relation between the shared ($\kappa_{1}^{t}\wedge \kappa_{2}^{t}$) and redundant ($\kappa_{1}^{t}⊓\kappa_{2}^{t}$) decision regions. The channel $\kappa_{1}^{t}$ consists of the vectors ${\vec{a}}_{x}$, and the channel $\kappa_{2}^{t}$ consists of the vectors ${\vec{b}}_{x}$.

## Data Availability

Not applicable.

## Abbreviations

The following abbreviations are used:

PID	Partial Information Decomposition
ROC	Receiver Operating Characteristic
TPR	True-Positive Rate ($\bar{\beta }$)
FPR	False-Positive Rate (α)

We use the following notation conventions:	
*T*, T, *t*, $T^{t}$	*T* (upper case) represents the target variable with an event *t* (lower case) of its event space (calligraphic), t∈T. $T^{t}$ represents a pointwise (binary) target variable which takes state one if T=t and state two if T≠t ($T^{t}$ represents the one-vs-rest encoding of state *t*);
V, $V_{i}$, $V_{i}$, *v*	V represents a set of visible/observable/predictor variables $V_{i}$ with $v\in V_{i}$;
$S_{i},S_{i}$	*sources* represent a set of visible variables, where the index *i* lists the contained visible variables, such as $S_{12}=\left\{V_{1},V_{2}\right\}$. The event $s\in S_{i}$ corresponds to an event of the corresponding joint variable, e.g., $\left(V_{1},V_{2}\right)$.
We represent channels (κ, λ) as row stochastic matrices with the following indexing:	
P	represents a permutation matrix;
$\kappa_{i}$	represents a channel from the target to a source $T\overset{\kappa_{i}}{\to }S_{i}$ using the joint distribution of the variables within the source, such as $T\overset{\kappa_{12}}{\to }\left(V_{1},V_{2}\right)$;
$\kappa_{i}^{t}$	represents a pointwise channel from the target to a source $T^{t}\overset{\kappa_{i}^{t}}{\to }S_{i}$, such as $T^{t}\overset{\kappa_{12}^{t}}{\to }\left(V_{1},V_{2}\right)$;
$Z_{\kappa_{i}^{t}}$	binary input channels $\kappa_{i}^{t}$ can be represented as (row) stochastic matrix, which contain a likelihood vector ${\vec{v}}_{s}=\left(\begin{array}{c} p(S_{i}=s|T=t)\\ p(S_{i}=s|T\neq t) \end{array}\right)$ for each state $s\in S_{i}$. $Z_{\kappa_{i}^{t}}$ represents the zonotope for this set of vectors;
$\kappa_{1}^{t}\vee \kappa_{2}^{t}$	represents the binary input channel corresponding to the convex hull of $Z_{\kappa_{1}^{t}}$ and $Z_{\kappa_{2}^{t}}$ (Blackwell order joint of binary input channels $\kappa_{1}^{t}\vee \kappa_{2}^{t}\equiv \kappa_{1}^{t}⊔\kappa_{2}^{t}$);
$\kappa_{1}^{t}\wedge \kappa_{2}^{t}$	represents the meet element for constructing a distributive lattice with the joint operator $\kappa_{1}^{t}\vee \kappa_{2}^{t}$;
$\kappa_{1}^{t}⊓\kappa_{2}^{t}$	represents the binary input channel corresponding to the intersection of $Z_{\kappa_{1}^{t}}$ and $Z_{\kappa_{2}^{t}}$ (Blackwell order meet of binary input channels);
α, β	*atoms* represent an expression of random variables with the operators (∨/∧). In Section 2.2 and Section 4, they represent sets of sources;
$\alpha^{t}$, $\beta^{t}$	represent an expression of pointwise channels with the operators (∨/∧);
$\alpha^{\delta t}$, $\beta^{\delta t}$	represent a partial pointwise channel corresponding to $\alpha^{t}$.
We use the following convention for operations, functions and brackets:	
$P_{1}\left(\cdot \right)$	represents the power set without the empty set;
$\left\{V_{1},V_{2}\right\}$	curly brackets with comma separation represent a set;
$\left[\begin{array}{cc} M_{1} & M_{2} \end{array}\right]$	square brackets without comma separation represent a matrix, and the listing of matrices in this manner represents their concatenation;
q([α;β])	square brackets with semicolon separation are used to refer to the bi-valuation b(α,β) of a consistent lattice valuation q(α). In a similar manner to Knuth [13], we use the notation q([α;β])≡b(α,β);
(α;β)	round brackets with semicolon separation represent an element of a Cartesian product $L_{1}\times L_{2}$, where $\alpha \in L_{1}$ and $\beta \in L_{2}$;
f〈L〉	angled brackets indicate that a function *f* shall be mapped to each element of the set L. We may nest this notation, such as f〈〈L〉〉, to indicate a map to each element of the sets within L;
α	False-Positive Rate, type I error;
$\bar{\beta }$	True-Positive Rate, 1− type II error.
We distinguish between a *joint channel* $T\overset{\kappa_{12}}{\to }\left(V_{1},V_{2}\right)$ and the *joint of two channels* $\kappa_{1}\vee \kappa_{2}$. To avoid confusion, we write the first case as “joint channel (κ)” and the latter case as “joint of channels ($\kappa_{i}\vee \kappa_{j}$)” throughout this work.	

## Appendix A

The considered lattice relates the meet and joint elements (∧/∨) through an inclusion–exclusion principle. Here, the partial contribution for the joint of any two incomparable elements $\left(\alpha^{t},\beta^{t}\in B^{t}\left(V\right),\alpha^{t}\vee \beta^{t}≁\alpha^{t}\mathrm{and}\alpha^{t}\vee \beta^{t}≁\beta^{t}\right)$ shall be zero, which is indicated using a gray font in Figure A1.

## Appendix B

This section demonstrates that the defined meet and joint operators of Section 3.2 provide a distributive lattice under the defined equivalence relation (∼, Equation (10)).

**Lemma** **A1.**
*The meet and joint operators (∧, ∨) define a distributive lattice for a set of channels under the defined equivalence relation (∼).*

**Proof.** The definitions of the meet and joint satisfy associativity, commutativity, idempotency, absorption and distributivity on channels under the defined equivalence relation:

1. *Idempotency:* $\kappa_{1}^{t}\vee \kappa_{1}^{t}∼\kappa_{1}^{t}$ and $\kappa_{1}^{t}\wedge \kappa_{1}^{t}∼\kappa_{1}^{t}$.
  $$\begin{array}{cc} \kappa_{1}^{t}\vee \kappa_{1}^{t} & ∼\kappa_{1}^{t}⊔\kappa_{1}^{t}∼\kappa_{1}^{t};\\ \kappa_{1}^{t}\wedge \kappa_{1}^{t} & ∼\left[\begin{array}{ccc} \kappa_{1}^{t} & \kappa_{1}^{t} & -\kappa_{1}^{t}\vee \kappa_{1}^{t} \end{array}\right]∼\left[\begin{array}{ccc} \kappa_{1}^{t} & \kappa_{1}^{t} & -\kappa_{1}^{t} \end{array}\right]∼\kappa_{1}^{t}. \end{array}$$
2. *Commutativity:* $\kappa_{1}^{t}\vee \kappa_{2}^{t}∼\kappa_{2}^{t}\vee \kappa_{1}^{t}$ and $\kappa_{1}^{t}\wedge \kappa_{2}^{t}∼\kappa_{2}^{t}\wedge \kappa_{1}^{t}$.
  $$\begin{array}{cc} \kappa_{1}^{t}\vee \kappa_{2}^{t} & ∼\kappa_{1}^{t}⊔\kappa_{2}^{t}∼\kappa_{2}^{t}⊔\kappa_{1}^{t}∼\kappa_{2}^{t}\vee \kappa_{1}^{t};\\ \kappa_{1}^{t}\wedge \kappa_{2}^{t} & ∼\left[\begin{array}{ccc} \kappa_{1}^{t} & \kappa_{2}^{t} & -\kappa_{1}^{t}\vee \kappa_{2}^{t} \end{array}\right]∼\left[\begin{array}{ccc} \kappa_{2}^{t} & \kappa_{1}^{t} & -\kappa_{2}^{t}\vee \kappa_{1}^{t} \end{array}\right]∼\kappa_{2}^{t}\wedge \kappa_{1}^{t}. \end{array}$$
3. *Associativity:* $\kappa_{1}^{t}\vee \left(\kappa_{2}^{t}\vee \kappa_{3}^{t}\right)∼\left(\kappa_{1}^{t}\vee \kappa_{2}^{t}\right)\vee \kappa_{3}^{t}$ and $\kappa_{1}^{t}\wedge \left(\kappa_{2}^{t}\wedge \kappa_{3}^{t}\right)∼\left(\kappa_{1}^{t}\wedge \kappa_{2}^{t}\right)\wedge \kappa_{3}^{t}$.
  $$\begin{array}{cc} \kappa_{1}^{t}\vee \left(\kappa_{2}^{t}\vee \kappa_{3}^{t}\right) & ∼\kappa_{1}^{t}⊔\left(\kappa_{2}^{t}⊔\kappa_{3}^{t}\right)∼\left(\kappa_{1}^{t}⊔\kappa_{2}^{t}\right)⊔\kappa_{3}^{t}∼\left(\kappa_{1}^{t}\vee \kappa_{2}^{t}\right)\vee \kappa_{3}^{t};\\ \kappa_{1}^{t}\wedge \left(\kappa_{2}^{t}\wedge \kappa_{3}^{t}\right) & ∼\left[\begin{array}{ccccccc} \kappa_{1}^{t} & \kappa_{2}^{t} & \kappa_{3}^{t} & -\kappa_{1}^{t}\vee \kappa_{2}^{t} & -\kappa_{1}^{t}\vee \kappa_{3}^{t} & -\kappa_{2}^{t}\vee \kappa_{3}^{t} & \kappa_{1}^{t}\vee \kappa_{2}^{t}\vee \kappa_{3}^{t} \end{array}\right]\\ \; & ∼\left[\begin{array}{ccccccc} \kappa_{3}^{t} & \kappa_{2}^{t} & \kappa_{1}^{t} & -\kappa_{3}^{t}\vee \kappa_{2}^{t} & -\kappa_{3}^{t}\vee \kappa_{1}^{t} & -\kappa_{2}^{t}\vee \kappa_{1}^{t} & \kappa_{3}^{t}\vee \kappa_{2}^{t}\vee \kappa_{1}^{t} \end{array}\right]\\ \; & ∼\kappa_{3}^{t}\wedge \left(\kappa_{2}^{t}\wedge \kappa_{1}^{t}\right)∼\left(\kappa_{1}^{t}\wedge \kappa_{2}^{t}\right)\wedge \kappa_{3}^{t}. \end{array}$$
4. *Absorption:* $\kappa_{1}^{t}\wedge \left(\kappa_{1}^{t}\vee \kappa_{2}^{t}\right)∼\kappa_{1}^{t}$ and $\kappa_{1}^{t}\vee \left(\kappa_{1}^{t}\wedge \kappa_{2}^{t}\right)∼\kappa_{1}^{t}$.
  $$\begin{array}{cc} \kappa_{1}^{t}\wedge \left(\kappa_{1}^{t}\vee \kappa_{2}^{t}\right) & ∼\left[\begin{array}{ccc} \kappa_{1}^{t} & \kappa_{1}^{t}\vee \kappa_{2}^{t} & -\kappa_{1}^{t}\vee \kappa_{1}^{t}\vee \kappa_{2}^{t} \end{array}\right]\\ \; & ∼\left[\begin{array}{ccc} \kappa_{1}^{t} & \kappa_{1}^{t}\vee \kappa_{2}^{t} & -\kappa_{1}^{t}\vee \kappa_{2}^{t} \end{array}\right]∼\kappa_{1}^{t};\\ \kappa_{1}^{t}\vee \left(\kappa_{1}^{t}\wedge \kappa_{2}^{t}\right) & ∼\left[\begin{array}{ccc} \kappa_{1}^{t} & \kappa_{1}^{t}\wedge \kappa_{2}^{t} & -\kappa_{1}^{t}\wedge \kappa_{1}^{t}\wedge \kappa_{2}^{t} \end{array}\right]\\ \; & ∼\left[\begin{array}{ccc} \kappa_{1}^{t} & \kappa_{1}^{t}\wedge \kappa_{2}^{t} & -\kappa_{1}^{t}\wedge \kappa_{2}^{t} \end{array}\right]∼\kappa_{1}^{t}. \end{array}$$
5. *Distributivity:* $\kappa_{1}^{t}\vee \left(\kappa_{2}^{t}\wedge \kappa_{3}^{t}\right)∼\left(\kappa_{1}^{t}\vee \kappa_{2}^{t}\right)\wedge \left(\kappa_{1}^{t}\vee \kappa_{3}^{t}\right)$ and $\kappa_{1}^{t}\wedge \left(\kappa_{2}^{t}\vee \kappa_{3}^{t}\right)∼\left(\kappa_{1}^{t}\wedge \kappa_{2}^{t}\right)\vee \left(\kappa_{1}^{t}\wedge \kappa_{3}^{t}\right)$.
  $$\begin{array}{cc} \kappa_{1}^{t}\vee \left(\kappa_{2}^{t}\wedge \kappa_{3}^{t}\right) & ∼\left[\begin{array}{ccc} \kappa_{2}^{t}\wedge \kappa_{3}^{t} & \kappa_{1}^{t} & -\kappa_{1}^{t}\wedge \left(\kappa_{2}^{t}\wedge \kappa_{3}^{t}\right) \end{array}\right]\\ \; & ∼\left[\begin{array}{ccccccc} \kappa_{2}^{t}\wedge \kappa_{3}^{t} & -\kappa_{2}^{t} & -\kappa_{3}^{t} & \kappa_{1}^{t}\vee \kappa_{2}^{t} & \kappa_{1}^{t}\vee \kappa_{3}^{t} & \kappa_{2}^{t}\vee \kappa_{3}^{t} & -\kappa_{1}^{t}\vee \kappa_{2}^{t}\vee \kappa_{3}^{t} \end{array}\right]\\ \; & ∼\left[\begin{array}{ccccc} -\kappa_{2}^{t}\vee \kappa_{3}^{t} & \kappa_{1}^{t}\vee \kappa_{2}^{t} & \kappa_{1}^{t}\vee \kappa_{3}^{t} & \kappa_{2}^{t}\vee \kappa_{3}^{t} & -\kappa_{1}^{t}\vee \kappa_{2}^{t}\vee \kappa_{3}^{t} \end{array}\right]\\ \; & ∼\left[\begin{array}{ccc} \kappa_{1}^{t}\vee \kappa_{2}^{t} & \kappa_{1}^{t}\vee \kappa_{3}^{t} & -\kappa_{1}^{t}\vee \kappa_{2}^{t}\vee \kappa_{3}^{t} \end{array}\right]\\ \; & ∼\left[\begin{array}{ccc} \kappa_{1}^{t}\vee \kappa_{2}^{t} & \kappa_{1}^{t}\vee \kappa_{3}^{t} & -\left(\kappa_{1}^{t}\vee \kappa_{2}^{t}\right)\vee \left(\kappa_{1}^{t}\vee \kappa_{3}^{t}\right) \end{array}\right]\\ \; & ∼\left(\kappa_{1}^{t}\vee \kappa_{2}^{t}\right)\wedge \left(\kappa_{1}^{t}\vee \kappa_{3}^{t}\right);\\ \kappa_{1}^{t}\wedge \left(\kappa_{2}^{t}\vee \kappa_{3}^{t}\right) & ∼\left[\begin{array}{ccc} \kappa_{2}^{t}\vee \kappa_{3}^{t} & \kappa_{1}^{t} & -\kappa_{1}^{t}\vee \left(\kappa_{2}^{t}\vee \kappa_{3}^{t}\right) \end{array}\right]\\ \; & ∼\left[\begin{array}{ccccccc} \kappa_{2}^{t}\vee \kappa_{3}^{t} & -\kappa_{2}^{t} & -\kappa_{3}^{t} & \kappa_{1}^{t}\wedge \kappa_{2}^{t} & \kappa_{1}^{t}\wedge \kappa_{3}^{t} & \kappa_{2}^{t}\wedge \kappa_{3}^{t} & -\kappa_{1}^{t}\wedge \kappa_{2}^{t}\wedge \kappa_{3}^{t} \end{array}\right]\\ \; & ∼\left[\begin{array}{ccccc} -\kappa_{2}^{t}\wedge \kappa_{3}^{t} & \kappa_{1}^{t}\wedge \kappa_{2}^{t} & \kappa_{1}^{t}\wedge \kappa_{3}^{t} & \kappa_{2}^{t}\wedge \kappa_{3}^{t} & -\kappa_{1}^{t}\wedge \kappa_{2}^{t}\wedge \kappa_{3}^{t} \end{array}\right]\\ \; & ∼\left[\begin{array}{ccc} \kappa_{1}^{t}\wedge \kappa_{2}^{t} & \kappa_{1}^{t}\wedge \kappa_{3}^{t} & -\left(\kappa_{1}^{t}\wedge \kappa_{2}^{t}\right)\wedge \left(\kappa_{1}^{t}\wedge \kappa_{3}^{t}\right) \end{array}\right]\\ \; & ∼\left(\kappa_{1}^{t}\wedge \kappa_{2}^{t}\right)\vee \left(\kappa_{1}^{t}\wedge \kappa_{3}^{t}\right). \end{array}$$

   □

## Appendix C

This section demonstrates the quantification of a small example and proves that the function *f* of Equation (19) creates a *consistent valuation* $\alpha^{t}\wedge \beta^{t}∼\beta^{t}\Rightarrow f\left(\beta^{t}\right)\leq f\left(\alpha^{t}\right)$ for the pointwise lattice $\left(B^{t}\left(V\right),\wedge ,\vee \right)$.

The convexity of the function $r(\vec{v})$ results, in combination with the property that $r\left(\ell \vec{v}\right)=\ell r\left(\vec{v}\right)$ with ℓ∈R, in a triangle inequality, as shown in Equation (A1). This ensures that Blackwell superior channels obtain a larger quantification result and thus the non-negativity of channels: $f\left(\kappa^{t}⊔\lambda^{t}\right)\geq f\left(\kappa^{t}\right)\geq f\left(\left[\begin{array}{c} 1\\ 1 \end{array}\right]\right)=0$.

(A1) $$\begin{array}{ccc} r(t{\vec{v}}_{1}+\left(1-t\right){\vec{v}}_{2}) & \leq tr\left({\vec{v}}_{1}\right)+\left(1-t\right)r\left({\vec{v}}_{2}\right) & \;\left(\mathrm{convexity},0\leq t\leq 1\right)\\ r({\vec{v}}_{1}+{\vec{v}}_{2}) & \leq r\left({\vec{v}}_{1}\right)+r\left({\vec{v}}_{2}\right) & \;\left(\mathrm{using}t=0.5\mathrm{and}r\left(\ell \vec{v}\right)=\ell r\left(\vec{v}\right)\right) \end{array}$$

To provide an intuition for the meet operator with a minimal example and highlight its relation to the intersection of zonotopes (redundant region), consider the two channels $\kappa_{1}^{t}$ and $\kappa_{2}^{t}$ of Equation (A2) and as visualized in Figure A2. To simplify the notation, we use the property $\left[\begin{array}{c} \left(\left(1+\ell \right){\vec{v}}_{1}\right) \end{array}\right]∼\left[\begin{array}{cc} \left({\vec{v}}_{1}\right) & \;(\ell {\vec{v}}_{1}) \end{array}\right]$ to differentiate the vectors ${\vec{a}}_{2}$ and ${\vec{a}}_{3}$ as well as ${\vec{b}}_{1}$ and ${\vec{b}}_{2}$.

(A2) $$\begin{array}{cc} \kappa_{1}^{t} & ∼\left[\begin{array}{ccc} \left({\vec{a}}_{1}\right) & \left({\vec{a}}_{2}\right) & \left({\vec{a}}_{3}\right) \end{array}\right]\\ \kappa_{2}^{t} & ∼\left[\begin{array}{ccc} \left({\vec{b}}_{1}\right) & \left({\vec{b}}_{2}\right) & \left({\vec{b}}_{3}\right) \end{array}\right]\\ \kappa_{1}^{t}\vee \kappa_{2}^{t} & ∼\left[\begin{array}{ccc} \left({\vec{a}}_{1}\right) & \left({\vec{a}}_{2}+{\vec{b}}_{2}\right) & \left({\vec{b}}_{3}\right) \end{array}\right] \end{array}$$

The resulting shared and redundant element is shown in Equation (A3). Due to the construction of the meet element through an inclusion–exclusion principle with the joint, the meet element always contains the vectors which span the redundant decision region as the first component.

(A3) $$\begin{array}{cc} \kappa_{1}^{t}\wedge \kappa_{2}^{t} & ∼\left[\begin{array}{ccccc} \left({\vec{b}}_{1}\right) & \left({\vec{a}}_{3}\right) & \left({\vec{b}}_{2}\right) & \left({\vec{a}}_{2}\right) & -({\vec{a}}_{2}+{\vec{b}}_{2}) \end{array}\right]\\ \kappa_{1}^{t}⊓\kappa_{2}^{t} & ∼\left[\begin{array}{cc} \left({\vec{b}}_{1}\right) & \left({\vec{a}}_{3}\right) \end{array}\right] \end{array}$$

The second component of the meet element corresponds to the decision region of the joint, which is not part of either individual channel. This component is non-negative due to the triangle inequality.

(A4) $$0\;\leq \;f\left(\kappa_{1}^{t}\wedge \kappa_{2}^{t}\right)\;-\;f\left(\kappa_{1}^{t}⊓\kappa_{2}^{t}\right)=r\left({\vec{a}}_{2}\right)+r\left({\vec{b}}_{2}\right)-r\left({\vec{a}}_{2}+{\vec{b}}_{2}\right)$$

The same argument applies to the meet for an arbitrary number of channels since the inclusion–exclusion principle with the joint elements ensures that the vectors spanning the redundant region are contained in the meet element, and the triangle inequality ensures non-negativity for the additional components.

**Lemma** **A2.**
*The function $f(\alpha^{t})$ is a (consistent) valuation $\alpha^{t}\wedge \beta^{t}∼\beta^{t}\Rightarrow f\left(\beta^{t}\right)\leq f\left(\alpha^{t}\right)$ on the pointwise lattice corresponding to $\left(B^{t}\left(V\right),\wedge ,\vee \right)$, as visualized in Appendix A.*

**Proof.** Let $S^{t}=\left\{\kappa_{1}^{t},\ldots ,\kappa_{a}^{t}\right\}$ represent a set of pointwise channels. The meet element (${⋀}_{\lambda^{t}\in S^{t}}\lambda^{t}$) is constructed through an inclusion–exclusion principle with the joint (convex hull). This ensures that the set of vectors spanning the zonotope intersection (${⊓}_{\lambda^{t}\in S^{t}}\lambda^{t}$) is contained within the meet element. Additionally, the meet contains a second component that is ensured to be positive from the triangle inequality of *r*: $f\left({⋀}_{\lambda^{t}\in S^{t}}\lambda^{t}\right)\geq f($ ${⊓}_{\lambda^{t}\in S^{t}}\lambda^{t})$. Since the joint operator is closed on channels and is distributive, we can introduce a channel to enforce a minimal redundant decision region between the channels: $f\left(\kappa_{0}^{t}\right)\leq f($ ${⊓}_{\lambda^{t}\in S^{t}}\;\kappa_{0}^{t}⊔\lambda^{t})\leq f\left({⋀}_{\lambda^{t}\in S^{t}}\;\kappa_{0}^{t}\vee \lambda^{t}\right)=f\left(\kappa_{0}^{t}\vee {⋀}_{\lambda^{t}\in S^{t}}\;\lambda^{t}\right)$. Applying the sum-rule shows that $f\left(\kappa_{0}^{t}\wedge {⋀}_{\lambda^{t}\in S^{t}}\;\lambda^{t}\right)\leq f\left({⋀}_{\lambda^{t}\in S^{t}}\;\lambda^{t}\right)$. We again make use of the distributive property, which allows writing any expression $\alpha^{t}$ into a conjunctive normal form. Since the joint operator is closed for channels, any expression $\alpha^{t}$ can be represented as meet for a set of channels $\alpha^{t}∼{⋀}_{\lambda^{t}\in \left\{\kappa_{p_{1}}^{t},\ldots ,\kappa_{p_{i}}^{t}\right\}}\;\lambda^{t}$. This demonstrates that the obtained inequality of the meet operator on channels also applies to atoms $f\left(\alpha^{t}\wedge \beta^{t}\right)\leq f\left(\alpha^{t}\right)$, such that $\alpha^{t}\wedge \beta^{t}∼\beta^{t}\Rightarrow f\left(\beta^{t}\right)\leq f\left(\alpha^{t}\right)$.    □

## Appendix D

The considered function $f_{p}\left(\kappa^{t}\right)$ of Section 3.2 takes the sum of a convex function. The Hessian matrix $H_{r}$ of the function $r_{p}\left(x,y\right)=x\log_{b}\left(\frac{x}{px+(1-p)y}\right)$ is positive-semidefinite in the required domain (symmetric and its eigenvalues $e_{1}$ and $e_{2}$ are greater than or equal to zero for x>0 and b>1).

(A5) $$\begin{array}{cc} H_{r} & =\frac{1}{\log(b)}\left[\begin{array}{cc} \frac{{\left(p-1\right)}^{2}y^{2}}{x{\left(px+\left(1-p\right)y\right)}^{2}} & -\frac{{\left(p-1\right)}^{2}y}{{\left(px+\left(1-p\right)y\right)}^{2}}\\ -\frac{{\left(p-1\right)}^{2}y}{{\left(px+\left(1-p\right)y\right)}^{2}} & \frac{{\left(p-1\right)}^{2}x}{{\left(px+\left(1-p\right)y\right)}^{2}} \end{array}\right]\\ e_{1} & =0\\ e_{2} & =\frac{{\left(p-1\right)}^{2}\left(x^{2}+y^{2}\right)}{x\log\left(b\right){\left(px+\left(1-p\right)y\right)}^{2}} \end{array}$$

## Appendix E

We use the examples of Finn and Lizier [20] since they provided an extensive discussion of their motivation. We compare our decomposition results to $I_{\min}$ of Williams and Beer [5] and $I^{\pm }$ of Finn and Lizier [20]. Examples with two sources are additionally compared to $I^{\mathrm{BROJA}}$ of Bertschinger et al. [10] and Griffith and Koch [11]. We notate the results for shared information $S(V_{1},V_{2};T)$, unique information $U(V_{x};T)$ and synergetic/complementing information $C(V_{1},V_{2};T)$. We use the implementation of $I_{\min}$, $I^{\mathrm{BROJA}}$ and $I^{\pm }$ provided by the dit Python package for discrete information theory [24].

Notice that our approach is identical to Williams and Beer [5] if one of the variables is pointwise (for each $T^{t}$, not necessarily the same one each time) Blackwell superior to another, and that our approach is equal to Bertschinger et al. [10] and Griffith and Koch [11] for two visible variables at a *binary* target variable.

We would like to highlight Table A1 for the difference in our approach to Williams and Beer [5]. This is an arbitrary example, where the variables $V_{1}$ and $V_{2}$ are not Blackwell superior to each other from the perspective of $T^{t}$, as visualized in Figure 6. For highlighting the difference in our approach to Bertschinger et al. [10] and Griffith and Koch [11], we require an example where the target variable is not binary, such as the two-bit copy example in Table A2.

It can be seen that our approach does not satisfy the identity axiom of Harder et al. [8]. This axiom demands the decomposition of the two-bit-copy example (Table A2) to both variables providing one bit unique information and demands negative partial contributions in the three-bit even-parity example (Figure A3) [8,20].

**Table A1:** Two incomparable channels (visualized in Section 3.1). The table highlights the difference in our approach to Williams and Beer [5] while being identical to Bertschinger et al. [10] since the target variable is binary.

(a) Distribution				(b) Results				
$V_{1}$	$V_{2}$	T	**Pr**	**Method**	$S(V_{1},V_{2};T)$	$U(V_{1};T)$	$U(V_{2};T)$	$C(V_{1},V_{2};T)$
0	0	0	0.0625	${\hat{I}}_{T}\cdot H\left(T\right)$	0.1196	0.0272	0.0716	0.1205
0	0	1	0.3	$I_{\min}$ [5]	0.1468	0	0.0444	0.1477
1	0	0	0.0375	$I^{\pm }$ [20]	0.3214	−0.1746	−0.1302	0.3223
1	0	1	0.05	$I^{\mathrm{BROJA}}$ [10,11]	0.1196	0.0272	0.0716	0.1205
0	1	0	0.1875					
0	1	1	0.15					
1	1	0	0.2125					

**Table A2:** Two-bit-copy (TBC) example. The results of our approach differ from Bertschinger et al. [10] and Griffith and Koch [11] since the target variable is not binary.

(a) Distribution				(b) Results				
$V_{1}$	$V_{2}$	T	**Pr**	**Method**	$S(V_{1},V_{2};T)$	$U(V_{1};T)$	$U(V_{2};T)$	$C(V_{1},V_{2};T)$
0	0	0	1/4	${\hat{I}}_{T}\cdot H\left(T\right)$	1	0	0	1
0	1	1	1/4	$I_{\min}$ [5]	1	0	0	1
1	0	2	1/4	$I^{\pm }$ [20]	1	0	0	1
1	1	3	1/4	$I^{\mathrm{BROJA}}$ [10,11]	0	1	1	0

**Table A3:** XOR-gate (Xor) example. All compared measures provide the same results.

(a) Distribution				(b) Results				
$V_{1}$	$V_{2}$	T	**Pr**	**Method**	$S(V_{1},V_{2};T)$	$U(V_{1};T)$	$U(V_{2};T)$	$C(V_{1},V_{2};T)$
0	0	0	1/4	${\hat{I}}_{T}\cdot H\left(T\right)$	0	0	0	1
0	1	1	1/4	$I_{\min}$ [5]	0	0	0	1
1	0	1	1/4	$I^{\pm }$ [20]	0	0	0	1
1	1	0	1/4	$I^{\mathrm{BROJA}}$ [10,11]	0	0	0	1

**Table A4:** Pointwise unique (PwUnq) example. Our approach provides the same results as Williams and Beer [5] and Bertschinger et al. [10].

(a) Distribution				(b) Results				
$V_{1}$	$V_{2}$	T	**Pr**	**Method**	$S(V_{1},V_{2};T)$	$U(V_{1};T)$	$U(V_{2};T)$	$C(V_{1},V_{2};T)$
0	1	0	1/4	${\hat{I}}_{T}\cdot H\left(T\right)$	0.5	0	0	0.5
1	0	0	1/4	$I_{\min}$ [5]	0.5	0	0	0.5
0	2	1	1/4	$I^{\pm }$ [20]	0	0.5	0.5	0
2	0	1	1/4	$I^{\mathrm{BROJA}}$ [10,11]	0.5	0	0	0.5

**Table A5:** Redundant Error (RdnErr) example. Our approach provides the same results as Williams and Beer [5] and Bertschinger et al. [10].

(a) Distribution				(b) Results				
$V_{1}$	$V_{2}$	T	**Pr**	**Method**	$S(V_{1},V_{2};T)$	$U(V_{1};T)$	$U(V_{2};T)$	$C(V_{1},V_{2};T)$
0	0	0	3/8	${\hat{I}}_{T}\cdot H\left(T\right)$	0.189	0.811	0	0
1	1	1	3/8	$I_{\min}$ [5]	0.189	0.811	0	0
0	1	0	1/8	$I^{\pm }$ [20]	1	0	−0.811	0.811
1	0	1	1/8	$I^{\mathrm{BROJA}}$ [10,11]	0.189	0.811	0	0

**Table A6:** Unique (Unq) example. Our approach provides the same results as Williams and Beer [5] and Bertschinger et al. [10].

(a) Distribution				(b) Results				
$V_{1}$	$V_{2}$	T	**Pr**	**Method**	$S(V_{1},V_{2};T)$	$U(V_{1};T)$	$U(V_{2};T)$	$C(V_{1},V_{2};T)$
0	0	0	1/4	${\hat{I}}_{T}\cdot H\left(T\right)$	0	1	0	0
0	1	0	1/4	$I_{\min}$ [5]	0	1	0	0
1	0	1	1/4	$I^{\pm }$ [20]	1	0	−1	1
1	1	1	1/4	$I^{\mathrm{BROJA}}$ [10,11]	0	1	0	0

**Table A7:** And-gate (And) example. Our approach provides the same results as Williams and Beer [5] and Bertschinger et al. [10].

(a) Distribution				(b) Results				
$V_{1}$	$V_{2}$	T	**Pr**	**Method**	$S(V_{1},V_{2};T)$	$U(V_{1};T)$	$U(V_{2};T)$	$C(V_{1},V_{2};T)$
0	0	0	1/4	${\hat{I}}_{T}\cdot H\left(T\right)$	0.311	0	0	0.5
0	1	0	1/4	$I_{\min}$ [5]	0.311	0	0	0.5
1	0	0	1/4	$I^{\pm }$ [20]	0.561	−0.25	−0.25	0.75
1	1	1	1/4	$I^{\mathrm{BROJA}}$ [10,11]	0.311	0	0	0.5

## References

[1] Lizier J.T., Bertschinger N., Jost J., Wibral M. (2018). Information Decomposition of Target Effects from Multi-Source Interactions: Perspectives on Previous, Current and Future Work. Entropy.

[2] Wibral M., Finn C., Wollstadt P., Lizier J.T., Priesemann V. (2017). Quantifying Information Modification in Developing Neural Networks via Partial Information Decomposition. Entropy.

[3] Rassouli B., Rosas F.E., Gündüz D. (2020). Data Disclosure Under Perfect Sample Privacy. IEEE Trans. Inf. Forensics Secur..

[4] Rosas F.E., Mediano P.A.M., Rassouli B., Barrett A.B. (2020). An operational information decomposition via synergistic disclosure. J. Phys. Math. Theor..

[5] Williams P.L., Beer R.D. (2010). Nonnegative Decomposition of Multivariate Information. arXiv.

[6] Griffith V., Chong E.K.P., James R.G., Ellison C.J., Crutchfield J.P. (2014). Intersection Information Based on Common Randomness. Entropy.

[7] Bertschinger N., Rauh J., Olbrich E., Jost J., Gilbert T., Kirkilionis M., Nicolis G. (2013). Shared Information—New Insights and Problems in Decomposing Information in Complex Systems. Proceedings of the European Conference on Complex Systems 2012.

[8] Harder M., Salge C., Polani D. (2013). Bivariate measure of redundant information. Phys. Rev. E.

[9] Finn C. (2019). A New Framework for Decomposing Multivariate Information. Ph.D. Thesis.

[10] Bertschinger N., Rauh J., Olbrich E., Jost J., Ay N. (2014). Quantifying Unique Information. Entropy.

[11] Griffith V., Koch C. (2014). Quantifying Synergistic Mutual Information. Guided Self-Organization: Inception.

[12] Bertschinger N., Rauh J. The Blackwell Relation Defines No Lattice. Proceedings of the 2014 IEEE International Symposium on Information Theory.

[13] Knuth K.H. (2019). Lattices and Their Consistent Quantification. Ann. Der Phys..

[14] Blackwell D. (1953). Equivalent Comparisons of Experiments. The Annals of Mathematical Statistics.

[15] Fawcett T. (2006). An introduction to ROC analysis. Pattern Recognit. Lett..

[16] Schechtman E., Schechtman G. (2019). The relationship between Gini terminology and the ROC curve. Metron.

[17] Neyman J., Pearson E.S. (1933). IX. On the Problem of the Most Efficient Tests of Statistical Hypotheses. Philos. Trans. R. Soc. Lond. Ser. Contain. Pap. Math. Phys. Character.

[18] Mages T., Rohner C. (2023). Implementation: PID Quantifying Reachable Decision Regions. https://github.com/uu-core/pid-quantifying-reachable-decision-regions.

[19] Niu X., Quinn C.J. Information Flow in Markov Chains. Proceedings of the 2021 60th IEEE Conference on Decision and Control (CDC).

[20] Finn C., Lizier J.T. (2018). Pointwise Partial Information Decomposition Using the Specificity and Ambiguity Lattices. Entropy.

[21] Kolchinsky A. (2022). A Novel Approach to the Partial Information Decomposition. Entropy.

[22] Chicharro D., Panzeri S. (2017). Synergy and Redundancy in Dual Decompositions of Mutual Information Gain and Information Loss. Entropy.

[23] Rauh J., Bertschinger N., Olbrich E., Jost J. Reconsidering Unique Information: Towards a Multivariate Information Decomposition. Proceedings of the 2014 IEEE International Symposium on Information Theory.

[24] James R.G., Ellison C.J., Crutchfield J.P. (2018). dit: A Python package for discrete information theory. J. Open Source Softw..

